# Alpha-2-Macroglobulin and Signature Genes: Predictive Biomarkers for Prognosis and Immunotherapy in Clear Cell Renal Cell Carcinoma

**DOI:** 10.7150/jca.113242

**Published:** 2025-07-10

**Authors:** Ming Li, Xin Luo, Renyu Zhou, Minting Liu, Guang Wang, Xiaotan Zhang

**Affiliations:** 1 Department of Pathology, First Affiliated Hospital of Jinan University, School of Medicine, Jinan University, Guangzhou 510632, China; 2 International Joint Laboratory for Embryonic Development & Prenatal Medicine, Division of Histology and Embryology, School of Medicine, Jinan University, Guangzhou 510632, China; 3 Department of Urology, Sun Yat-sen University Cancer Center, Guangzhou, 510060, P.R. China.; 4 State Key Laboratory of Oncology in South China, Guangdong Provincial Clinical Research Center for Cancer, Sun Yat-sen University Cancer Center, Guangzhou, 510060, P. R. China; 5 Institute of Molecular and Medical Virology, School of Medicine, Jinan University, Guangzhou, 510630, China; 6 Key Laboratory of Regenerative Medicine of Ministry of Education, Jinan University, Guangzhou, 510632, China

**Keywords:** ccRCC, Immunotherapy response, Methylation, Prognosis prediction, Alpha-2-macroglobulin.

## Abstract

Alpha-2-macroglobulin (A2M) is a broad-spectrum protease inhibitor that plays a role in maintaining coagulation balance and immune regulation. Previous studies have demonstrated a strong association between A2M and various kidney diseases. However, little is known about the role of A2M in clear cell renal cell carcinoma (ccRCC). In this study, through pan-cancer analysis based on data from multiple public databases such as The Cancer Genome Atlas (TCGA) and Genotype-Tissue Expression (GTEx), a unique prognostic relationship between A2M and ccRCC was identified. A2M expression in three common RCCs and the prognosis were detected, which further proved that A2M was closely related to the prognosis of ccRCC, and the diagnostic value of A2M in ccRCC was determined. Additionally, the results found that A2M in ccRCC was regulated by methylation and affected vascularization and immune invasion. Subsequently, A2M-related genes were analyzed and 42 co-related gene expressions were identified in four public databases. Furthermore, a prognostic model [A2M gene-associated prognostic index (A2M-GPI)] composed of 7 genes [TIE1, VWF, TCF4, PTPRB, ICAM2, DOCK6, and RAMP3] was constructed using machine learning to predict the prognosis of ccRCC. Additionally, A2M-GPI combined with independent predictors (such as age, pathologic stage, and TNM stage) were used to create a survival Nomogram. This study is the first to systematically analyze the multiple mechanisms of A2M in the pathogenesis and progression of ccRCC. Machine learning was used to construct a prognostic model based on A2M to confirm that A2M is a valuable prognostic biomarker for ccRCC. Based on these findings, we created a publicly accessible website for its application (https://A2Mgpinomogram.shinyapps.io/ccRCC_prognosis_prediction/).

## Introduction

Renal cell carcinoma (RCC) is the most common malignancy in the kidneys and one of the leading causes of global death and morbidity, accounting for approximately 2% of cancer diagnoses and deaths worldwide 1. According to the statistics, approximately one-third of RCC patients have metastases at the time of diagnosis, with a high mortality rate and a 5-year survival rate of only 12% 2. RCC is a heterogeneous disease comprising various tumor types, with ccRCC accounting for the highest proportion of 75%-80%, followed by papillary RCC (pRCC) and chromophobe RCC (chRCC) 3. With the emergence and development of high-throughput sequencing technology, our understanding of the molecular drivers in the RCC subtype has also been improving. In the fifth edition of the classification of urogenital tumors published by WHO in 2022, in addition to the morphologically based classification, rare RCC categories defined by molecules (such as Fumarate hydratase-deficient RCC, Succinate dehydrogenase-deficient RCC, SMARCB1-deficient RCC, and ALK-rearranged RCC) has been added 4. The traditional clinical treatment methods of RCC are surgery, chemotherapy, and radiotherapy. Recently, novel therapeutic modalities including immunotherapy and targeted therapy have gradually emerged and been applied in clinical practice, such as vascular endothelial growth factor (VEGF) and tyrosine kinase inhibitor (TKI), yielding better therapeutic effects for patients 5. However, these therapies have some limitations, including high recurrence rate, serious side effects, drug resistance, and high price. Therefore, there is an urgent need to search for a new and highly accurate biomarker that can provide novel insights into treatment and prognosis prediction in RCC patients.

Alpha-2-macroglobulin (A2M) is a 725-kD high molecular weight homotetramer glycoprotein, which is mainly synthesized by the liver and has important biological activities. As a broad-spectrum protease inhibitor, A2M is involved in the maintenance of coagulation balance and immune regulation 6. In recent years, growing studies have revealed that A2M is tightly implicated in kidney diseases. For example, plasma A2M level is increased in patients with renal disease 7, 8. The expression level of A2M is correlated with the progression of focal segmental glomerulosclerosis and can serve as a predictor of treatment 9, 10. Additionally, it has been evidenced that A2M may play an important role in the pathogenesis of lupus nephritis and can be used as a biomarker for diagnosis or disease progression 11. However, few studies have been reported on A2M in ccRCC, and its clinical and prognostic significance in ccRCC remains unclear.

Therefore, this study aimed to analyze the expression of A2M in ccRCC and identify the relationship between A2M and the prognostic outcomes of ccRCC patients. Based on this research, we have developed a new prognostic A2M gene-associated prognostic index (A2M-GPI) to predict the therapeutic effects of A2M on the intervention and prognosis of ccRCC. This study offers new insights into utilizing A2M and its molecular signature genes to predict the prognosis and immunotherapy response in ccRCC.

## Materials and Methods

### Data preparation

RNAseq data and related clinical information of different tumors and normal tissues were provided by the Cancer Genome Atlas (TCGA) database and genotype-Tissue Expression (GTEx) database. RNAseq data and clinical information in TPM form GTEx and TCGA databases handled consistently by the Toil method were extracted from the UCSC XENA (https://xenabrowser.net/datapages/). We strictly followed the standard inclusion criteria provided by the TCGA database for ccRCC patients. All included cases were pathologically confirmed as ccRCC, and only high-quality, complete datasets were selected. Samples with incomplete information or ambiguous tumor classification were excluded. The specific inclusion and exclusion criteria were as follows: (a) Pathological confirmation of ccRCC; (b) Age ≥ 18 years; (c) Availability of both clinical data and gene expression profiles; (d) Presence of overall survival (OS) data; (e) Datasets containing at least 30 tumor samples with matched adjacent normal tissues; (f) Removal of technical replicates if necessary. Samples lacking survival time or survival status information, as well as those involving other cancer types, were excluded from this study. A total of 11069 pan-cancer samples were collected, among which ccRCC (normal tissue n = 90, cancer tissue n = 541), pRCC (normal tissue n = 58, cancer tissue n = 291), and chRCC (normal tissue n = 25, cancer tissue n = 65) data were used for subsequent data analysis. Moreover, the relationship between A2M gene expression and the survival and prognosis in ccRCC patients was investigated using Tumor Immune System Interaction Database (TISIDB, http://cis.hku.hk/TISIDB/), Gene Expression Profiling Interactive Analysis (GEPIA, http://gepia.cancer-pku.cn/), Kaplan Meier (https://kmplot.com/analysis/), and PROGgeneV2 (http://genomics.jefferson.edu/proggene/). A2M gene correlation analysis results in ccRCC patients were downloaded from cBioPortal (https://www.cbioportal.org/), LinkedOmics (https://www.linkedomics.org/), and GEPIA database. The Agilent microarray dataset and clinical characteristics of 101 ccRCC patients in the ArrayExpress (E-MTAB-1980) database and the mRNA transcriptomic data and clinical survival characteristics of 38 ccRCC patients (GSE29609) in the Gene Expression Omnibus (GEO) database were used for external validation of the model. Single-cell sequencing data (GSE152938) of ccRCC patients were extracted from the GEO database (https://www.ncbi.nlm.nih.gov/) and analyzed using the "Seurat V4" R package. The ccRCC tumor single-cell dataset (http://tisch.comp-genomics.org/) was obtained through Tumor Immune Single Cell Hub 2 (TISCH2).

### Human tumor tissue collection

A total of 70 cases of human RCC and 6 cases of paracancer paraffin-embedded tissues were used in this study. Samples in the tumor group were collected from patients diagnosed in the First Affiliated Hospital of Jinan University from 2016 to 2022, including 50 cases of ccRCC, 14 cases of pRCC, and 6 cases of chRCC (Table S1). After biopsy, the tumors were immediately washed 2 to 3 times with cold phosphate-buffered saline (PBS) to remove the blood and then fixed with liquid nitrogen or 4% paraformaldehyde for further analysis. The 4% paraformaldehyde-fixed tissue samples were subsequently embedded in paraffin. This study was approved by the Ethics Committees of the First Affiliated Hospital of Jinan University (NO.KY-2024-156) and conducted following the Declaration of Helsinki. Written informed consent was obtained from all individual participants included in the study.

### Prognostic value analysis

The "survival "and "survminer" R packages (v.3.2.1) were used for survival analysis and visualization of overall survival (OS), disease-specific survival (DSS), and progression-free survival (PFI) data 12. Kaplan-Meier curve and log-rank test were used to determine clinical prognostic features in cancer. Univariate and multivariate Cox regression analysis was performed using the "forestplot" and "survival" R packages to evaluate the association between A2M and survival prognosis in pancarcinoma.

### Gene methylation analysis

The A2M promoter methylation data of ccRCC patients was stored in Illumina Human Methylation 450K bead chip data in the TCGA database. After filtering out the duplicates and missing values of A2M sequencing data, the methylation data of a total of 319 patients were included. The RNAseq data in TCGA were converted from FPKM (fragments per kilobase per million) format into TPM (transcripts per million reads) for log2 conversion. Next, Spearman correlation visualization analysis was performed on A2M expression and cg08300930 methylation probe detection values using the "ggplot" package. The A2M methylation distribution and living conditions in ccRCC patients and normal people were evaluated using MethHC (https://awi.cuhk.edu.cn/~MethHC/) and UALCAN (https://ualcan.path.uab.edu/) web server. Beta values indicated the degree of DNA methylation, ranging from 0 (unmethylated) to 1 (fully methylated).

### Single gene difference analysis and functional enrichment

Data were re-grouped according to the median A2M expression level and subject to difference analysis using the "dplyr" R package and DESeq2 (v.1.26.0) package 13. Differentially expressed genes (DEGs) were screened with the threshold standard of *p* ≤ 0.05 and |log2FC| ≥ 1.5. Moreover, potential biological processes and pathways that DEGs may involve were identified using "clusterProfiler", "org.Hs.eg.db" (v. 3.10.0), "GOplot" (v. 1.0.2) R packages, and "c2.cp.kegg.v7.5.1.symbols.gmt" database.

Based on the "c2.cp.all.v7.5.1.symbols.gmt" database (R package "GSEA", "clusterProfiler", and "GSEABase"), GSEA used to analyze gene set changes in different pathways between the high-A2M expression group and low-A2M expression group.

### Single-cell RNAseq data analysis

Single-cell RNAseq data in the GSE152938 dataset were organized and analyzed using the "Seurat" R package. For each sample, cells with fewer than 20 unique molecular identifiers (UMIs) and cells with fewer than 200 or more than 5,000 expressing genes were eliminated. Additionally, dead cells with UMI of more than 5% of the mitochondrial genome were filtered out. After that, the top 2,000 highly variable genes were identified using the "Find Variable Features" function in Seurat and the "vst" method. The UMAP algorithm was used to reduce the dimensionality of data. Cell clusters were annotated using the Single R algorithm.

### Correlation analysis of tumor immune infiltration

The correlation between A2M expression and immune cell content in ccRCC patients was calculated using the ssGSEA algorithm in "GSVA" R package (v.1.34.0) 14. Characteristic markers were used to annotate 21 kinds of immune cells 15. The expression value of A2M in all kinds of ccRCC immune cells was analyzed utilizing the TISCH2 single-cell sequencing database. Additionally, the potential relationship between A2M expression and immunomodulators [such as immunoinhibitors, immunostimulators, chemokines, lymphocytes, major histocompatibility complex (MHC) molecules, and immune receptors] was evaluated through the TISIDB biological portal.

### Identification of gene associations and variation levels

The expression matrix of 541 ccRCC patients in the TCGA database was transformed using log2 (TPM+1), and samples with A2M expression ranking of 20-80% were filtered out. The "stat" package (v.3.6.3) was used for correlation analysis of RNA-seq data. The A2M correlation analysis results of the GEPIA database, cBioPortal, and LinkedOmics network server were extracted, and the relevant feature variables were screened with cor-spearman ≥ 0.6. The gene mutation data of ccRCC were downloaded from TCGA, and the mutations of 42 characteristic genes were analyzed and visualized using the "maftools" R package. Genes with high mutation frequency were displayed in histograms.

### Construction of A2M-related gene signatures

Univariate Cox regression was used to evaluate whether 42 A2M closely related genes had an impact on the survival state of ccRCC, and the adjusted threshold was *p* < 0.05. Least absolute shrinkage and selection operator (LASSO) regression and 10-fold cross-validation were performed using the "glmnet" R package to select the value of "lambda min", the smaller feature most relevant to the OS of patients in the TCGA-KIRC cohort, to further narrow down candidate genes and construct the most appropriate prognostic risk feature 16. Finally, the A2M-GPI for each patient was derived according to the following formula:



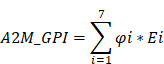




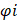
represents the risk coefficient, and *Ei* represents the expression of each gene. According to the median value of the final A2M-GPI score, ccRCC patients were allocated into the low A2M-GPI group and high A2M-GPI group. Principal component analysis (PCA) was performed using the "stats" package. Moreover, Kaplan-Meier analysis was performed on ccRCC samples with OS > 25 days using the "survival" and "survminer" packages to investigate the correlation between patient survival status and prognosis time and A2M-GPI scores.

### Establishment of the prognostic model

For a better application of A2M-GPI in the clinic, A2M-GPI was combined with other clinical features (gender, age, and T, N, M stage). Appropriate clinical features were selected by univariate post-multivariate Cox regression analysis to construct a prognostic nomogram. A nomogram model was established by integrating survival time, survival state, and 7 characteristic variables using the "rms" R package. Moreover, receiver operating characteristic (ROC) curve analysis was conducted utilizing the R packages "pROC" and "timeROC" (v.1.17.0.1) to obtain the area under the curve (AUC). ggplot was used for visualization. The R packages "caret" and "rmda" were used for calibration analysis of the nomogram and decision curve analysis (DCA) to evaluate the specificity and sensitivity of the prediction model. The 1-, 3 -, and 5-year survival of ccRCC patients could be accurately predicted by summing scores on clinical characteristic variables. The concordance index (C-index) value was used to indicate the accuracy of survival predictions. The dynamic nomogram was constructed using the "rsconnect" and the "DynNom" packages.

### Immunohistochemistry (IHC) analysis

The tissue sample was sectioned (4 μm) and baked (40 min) in a 65℃ oven. After dewaxing and rehydration using gradient ethanol, the tissue sections were subject to 15-min boiling in sodium citrate buffer (pH = 6.0) for antigen repair. Next, the sections were incubated (30 min) in 3% H_2_O_2_ to inhibit endogenous peroxidase activity, followed by rinsing in PBS. After that, the sections were incubated with polyclonal rabbit anti-A2M antibodies (1:100; ab109422, Abcam, UK) overnight in a shaker (4 °C). Subsequently, the sections were washed in PBS and incubated (room temperature, 3 h) with horseradish peroxidase (HRP)-labeled goat anti-rabbit IgG secondary antibody (1:400, EarthOx, USA) in a dark box, followed by visualization with DAB chromogenic reagent (Maixin Biotechnology, Fuzhou, China). The sections were then counter-stained (room temperature, 1 min) with hematoxylin, dehydrated in gradient alcohol, and sealed with neutral resin. The sections treated with PBS instead of A2M antibodies served as negative controls.

### Immunofluorescence staining

The sections were incubated (4°C, overnight) with primary anti-A2M antibody (1:100; ab109422, Abcam), Caveolin 1 (1:100; ab109422, Abcam), CD3 (1:100; ab109422, Abcam), and CD45 (1:100; ab109422, Abcam) in a shaker, followed by a further 2-h incubation (room temperature) with the corresponding Alexa Fluor 555 or 488 secondary antibody (1:1000, Invitrogen, USA) in a shaker. All sections undergoing immunofluorescence staining were counter-stained (room temperature, 1 h) with DAPI (1:1000, Invitrogen).

### DrugBank analysis

The DrugBank (https://go.drugbank.com/) database contains comprehensive molecular information, mechanisms, interactions, and targets of drugs 37. The Drugbank was used to analyze the pharmaco-transcriptomics of A2M.

### Statistical analysis

R software V.4.2.1 (https://www.r-project.org) was used for data mining and statistical analysis of some results. The survival curve was estimated using Kaplan-Meier survival analysis. The log-rank test was used to calculate the hazard ratio (HR) and 95% confidence interval (CI). Univariate and multivariate Cox regression analyses were performed to determine prognostic factors. The Mann-Whitney U test (Wilcoxon rank sum test) was used to show A2M expression values in unmatched samples, and the Kruskal-Wallis test was used to compare the differences in A2M-GPI and expression between different tumor stages. The correlation analysis was conducted using the Spearman test. Statistical mapping and analysis were performed using GraphPad Prism 7.0, and the results were shown as mean ± standard deviation (SD). The statistical differences between IHC groups were analyzed using a one-way analysis of variance (ANOVA). A *p*-value of < 0.05 was considered a statistically significant difference.

## Results

### Research workflow charts

**Figure 1:**
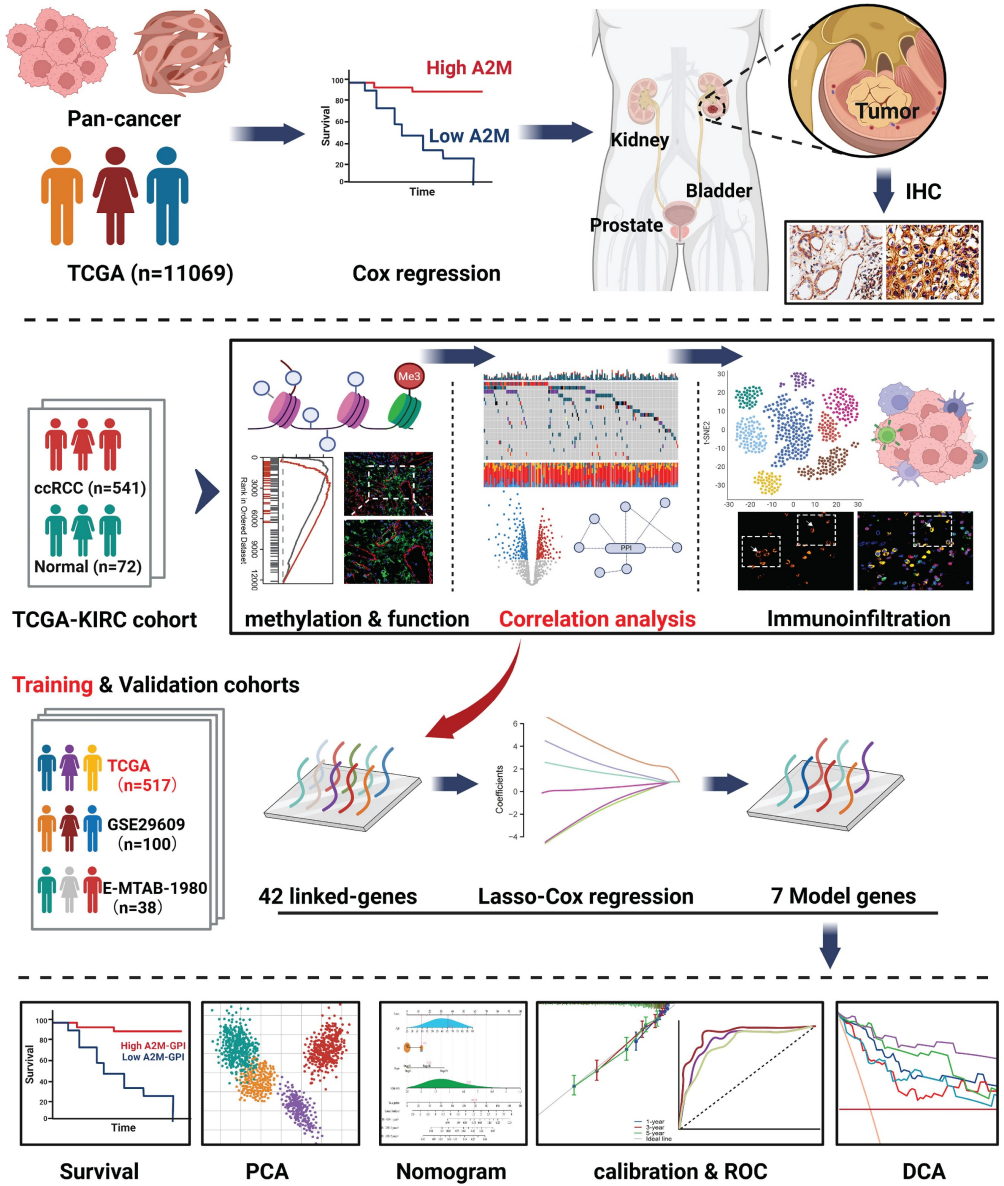
Exhibition of study workflow.

Through screening 11,069 pan-cancer patients in the TCGA database, a unique prognostic relationship between A2M and ccRCC was identified. Based on the function, correlation analysis results, and immunoinfiltration level of A2M in ccRCC, it's determined that the transcriptome data of 541 ccRCC, 291 pRCC, and 65 chRCC patients in the TCGA database and the single-cell RNA transcriptome data of 5 RCC patients from the GSE152938 database met the inclusion criteria. A2M protein expression level was verified in combination with tumor samples from 70 clinical RCC patients. Regarding the training and validation cohort in ccRCC, 517 patients in TCGA, 100 patients in GSE29609, and 38 patients in E-MTAB-1980 were selected. Through machine learning method, 42 related candidate feature genes were narrowed to 7 genes, and a prognostic model was constructed, evaluated, and validated in combination with clinical data. The flow chart of this study is shown in Figure 1.

### The prognostic effect of A2M on the OS and DSS in ccRCC in pan-cancer analysis

First, according to quantitative analysis results of A2M mRNA pan-cancer expression based on TIMER and TCGA databases, A2M showed significantly differential expression in most tumors compared with that in normal tissues (Figure S1). Among these tumors, A2M was remarkably downregulated in adrenocortical carcinoma (ACC), bladder urothelial carcinoma (BLCA), breast invasive carcinoma (BRCA), cervical squamous cell carcinoma and endocervical adenocarcinoma (CESC), cholangiocarcinoma (CHOL), colon adenocarcinoma (COAD), esophageal carcinoma (ESCA), kidney chromophobe (KICH), kidney renal papillary cell carcinoma (KIRP), lung adenocarcinoma (LUAD), lung squamous cell carcinoma (LUSC), ovarian serous cystadenocarcinoma (OV), prostate adenocarcinoma (PRAD), rectal adenocarcinoma (READ), uterine corpus endometrial carcinoma (UCEC), and uterine carcinosarcoma (UCS), whereas notably upregulated in diffuse large B-cell lymphoma (DLBC), glioblastoma multiforme (GBM), kidney renal clear cell carcinoma (KIRC), acute myeloid leukemia (LAML), brain low-grade glioma (LGG), pancreatic adenocarcinoma (PAAD), skin cutaneous melanoma (SKCM), stomach adenocarcinoma (STAD), testicular germ cell tumor (TGCT), and thymoma (THYM).

**Figure 2:**
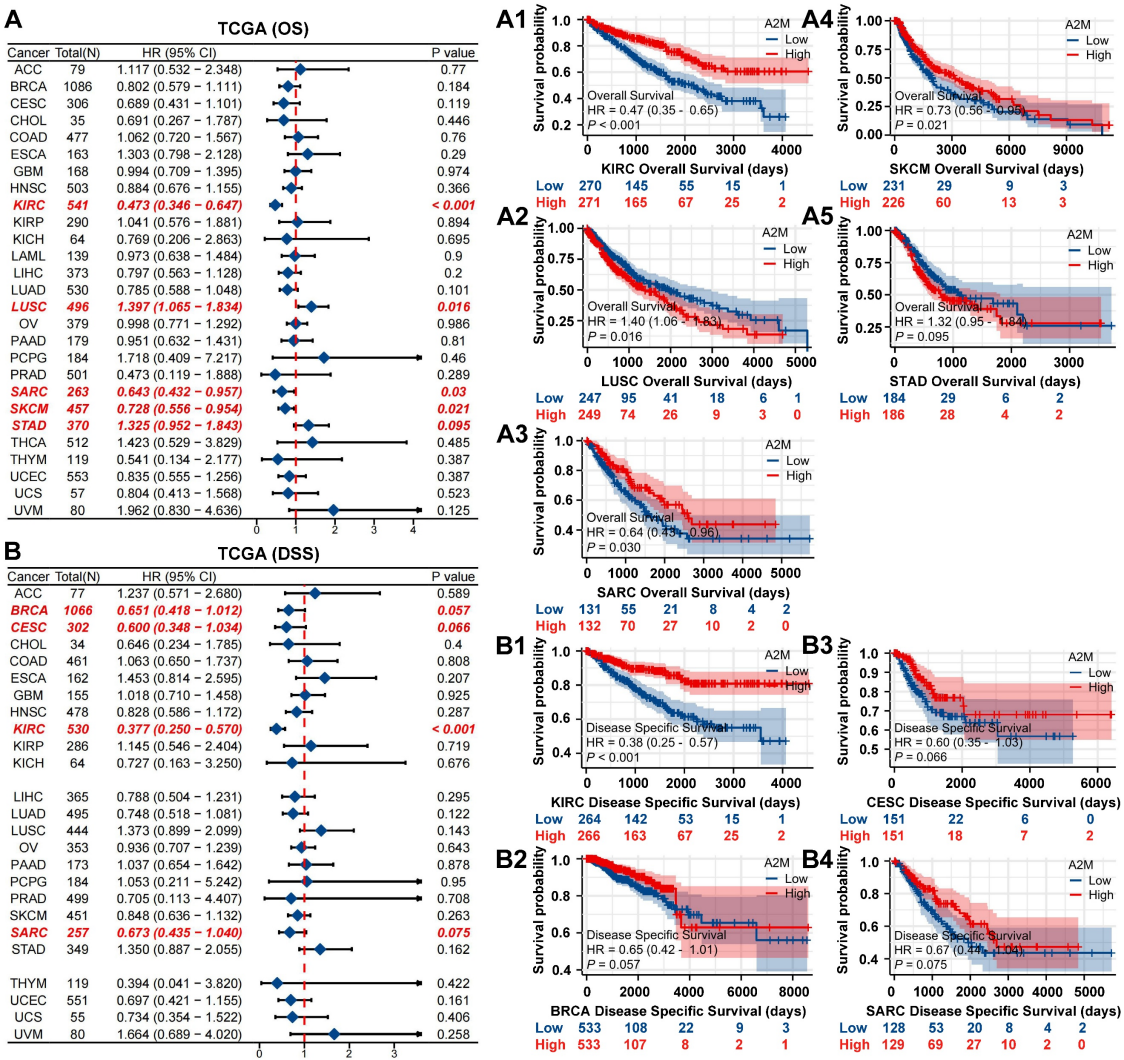
** Association between A2M expression level and pan-cancer OS and DSS. (A)** Forest map of multivariate Cox regression analysis of A2M and pan-cancer OS (red: p < 0.1); **(A1-A5)** Kaplan-Meier analysis of the OS of five tumors significantly affected by A2M; (**B**) forest map of multivariate Cox regression analysis of A2M and pan-cancer DSS (red mark: p < 0.1);** (B1-B4)** Kaplan-Meier analysis of the DSS of four tumors significantly affected by A2M.

The prognostic value of A2M in cancer was subsequently explored. In short, univariate Cox combined with multivariate Cox regression analysis (variables with a p-value of < 0.10 on univariate analysis were entered into a multivariate Cox analysis) was performed on the OS, DSS, and A2M expression of pancarcinoma in TCGA. The results showed that A2M expression was correlated with the OS prognosis of sarcomatoid cancer (SARC), SKCM, KIRC, LUSC, and STAD (p < 0.1) (Figure 2A). As indicated by Kaplan-Meier survival analysis results, high A2M expression was associated with a good overall prognostic OS in KIRC (Figure 2A1, p < 0.001), SARC (Figure 2A3, p = 0.030), SKCM (Figure 2A4, p = 0.021), and was related to a poor survival prognosis for OS in LUSC (Figure 2A2, p = 0.016). Additionally, according to Cox regression analysis results of DSS and A2M for pancarcinoma based on the TCGA database, differential expression of A2M was notably correlated with DSS prognosis of KIRC, SARC, BRCA, and CESC (p < 0.1) (Figure 2B). Moreover, Kaplan-Meier survival analysis results showed that the DSS of patients with high A2M expression in KIRC (Figure 2B1, p < 0.001) was dramatically better than that of patients with low A2M expression. Additionally, patients with BRCA (Figure 2B2, p = 0.057), CESC (Figure 2B3, p = 0.066), and SARC (Figure 2B4, p = 0.075) also showed a favorable prognostic trend for DSS. Although Cox proportional risk models of pan-cancer suggested that A2M was tightly associated with survival outcomes in multiple tumors, A2M was only significantly associated with both OS and DSS outcomes in KIRC (p < 0.001). This suggested that A2M was a potential novel prognostic biomarker in ccRCC, and its function in RCC needs to be further explored.

### 3.3 Diagnostic potential and prognostic effect of A2M in three renal carcinoma subtypes

**Figure 3:**
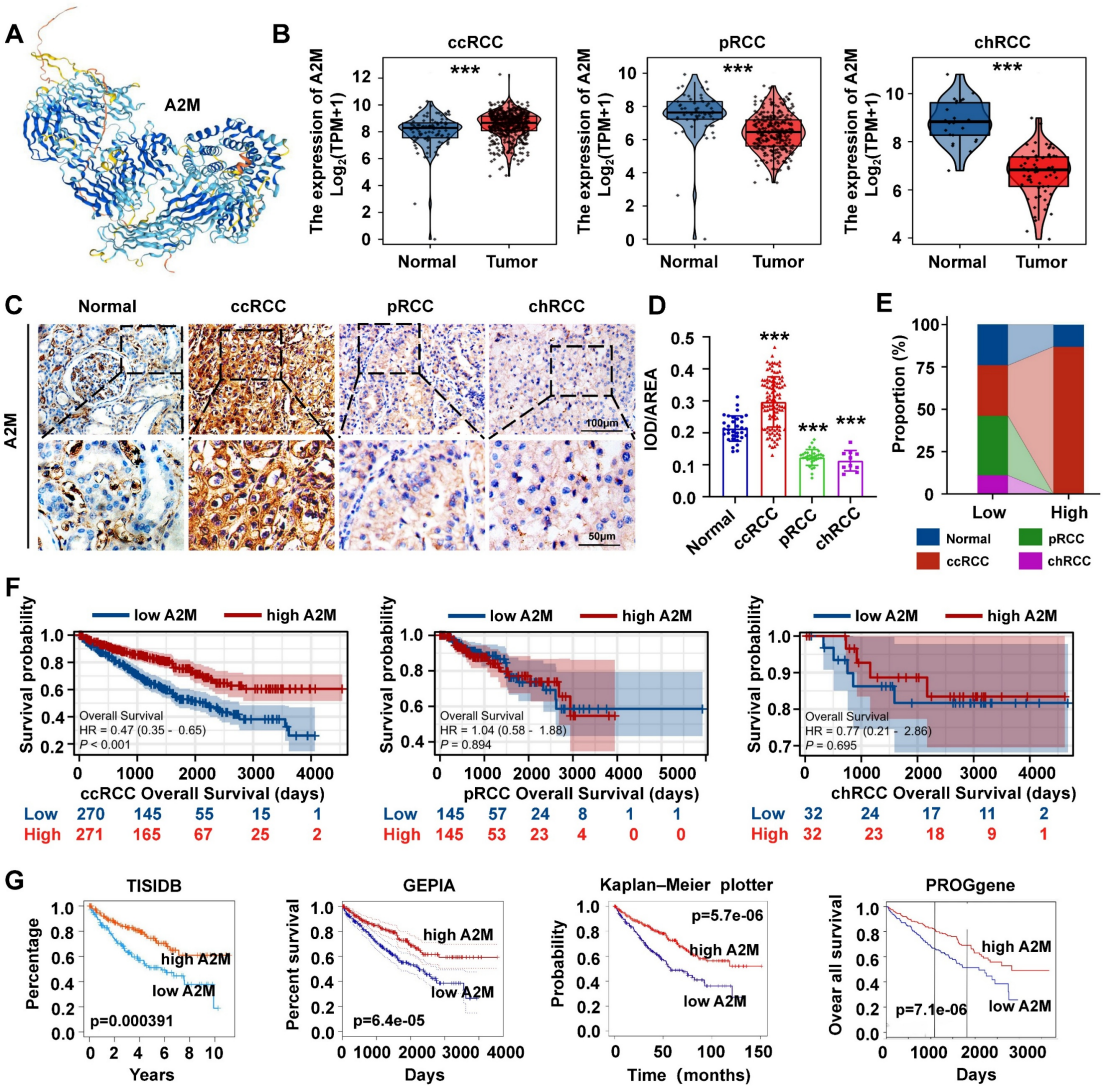
** A2M expression and prognosis analysis in ccRCC, pRCC, and chRCC. (A)** Protein structure map of A2M; **(B)** RNA expression levels of A2M in ccRCC, pRCC, chRCC, and normal samples in TCGA and GTEx databases; **(C)** IHC staining of A2M expression in tumor tissues of clinical ccRCC, pRCC, and chRCC patients; **(D)** statistical map of A2M expression level in C [mean optical density: integrated optical density (IOD)/area]; **(E)** redistribution according to the median mean optical density in D, and the superimposed histogram showed the distribution of tumor specimens of ccRCC, pRCC, and chRCC in different A2M expression levels; (**F**) Kaplan-Meier survival curve evaluation of the effect of A2M in TCGA on the prognosis of ccRCC, pRCC, and chRCC patients; (**G**) Kaplan-Meier survival curve evaluation of the effect of high or low A2M expression on the prognosis of ccRCC patients in TISIDB, GEPIA, Kaplan-Meier plotter, and PROGgene public databases; *p < 0.05, compared to normal; **p < 0.01, compared to normal; ***p < 0.001, compared to normal.

For further verifying the role of A2M in ccRCC, the protein structure of A2M was first predicted and synthesized using HPA (https://www.proteinatlas.org/) (Figure 3A). Subsequently, the TCGA and GTEx databases were used to analyze the transcriptome sequencing data of 897 TCGA tissues (541 ccRCC tissues, 291 pRCC tissues, and 65 chRCC tissues) and 173 normal tissues in the GTEx database. The results indicated differences in the expression trend of A2M in three types of RCC, with high A2M expression in ccRCC patients (p < 0.001) and low A2M expression in pRCC and chRCC patients (p < 0.001) (Figure 3B). Additionally, the clinical baseline data table of patient cohorts in TCGA-KIRC (Table 1), TCGA-KIRP (Table S2), and TCGA-KICH (Table S3) and the expression differences of A2M in different clinical stages indicated that A2M expression level had a significantly diagnostic potential for determination of disease clinical stage and evaluation of the main therapeutic effects (Figure S2). Subsequently, the A2M protein expression level was verified in 70 RCC tumor tissue samples from clinical patients of the First Affiliated Hospital of Jinan University and 6 control para-cancer tissue samples using IHC staining. In the control group, A2M was primarily localized to the cell membrane and cytoplasm, with positive staining observed in some glomeruli and tubules. In the ccRCC group, A2M was diffusely expressed in both the cell membrane and cytoplasm. In contrast, the pRCC and rRCC groups exhibited only weak A2M expression in these cellular compartments. The results revealed that the A2M protein level was consistent with its mRNA expression results (Figure 3C-D). According to regrouping and analysis results based on the average optical density of IHC staining of each sample, the superimposed bar graph showed that A2M high expression was mainly distributed in normal subjects (13.04%) and ccRCC patients (86.96%) (Figure 3E & Table S4). Next, with the median expression level of A2M mRNA as the cutoff point, the relationship between A2M mRNA expression level and survival time in different datasets was evaluated. It was found that ccRCC patients with high A2M expression in TCGA RNA-seq had higher OS levels (p < 0.001), with no significant effect on OS prognosis in pRCC (p = 0.894) and chRCC (p = 0.695) patients (Figure 3F). Similar results were also obtained in the verification of external databases including TISIDB (p = 0.000391), GEPIA (p = 6.4e-05), Kaplan-Meier plotter (p = 5.7e-06), and PROGgene (p = 7.1e-06) (Figure 3G). The above results further proved that A2M can be used as a potential diagnostic and prognostic biomarker for ccRCC.

### 3.4 Analysis of A2M methylation level and biological enrichment in ccRCC

**Figure 4:**
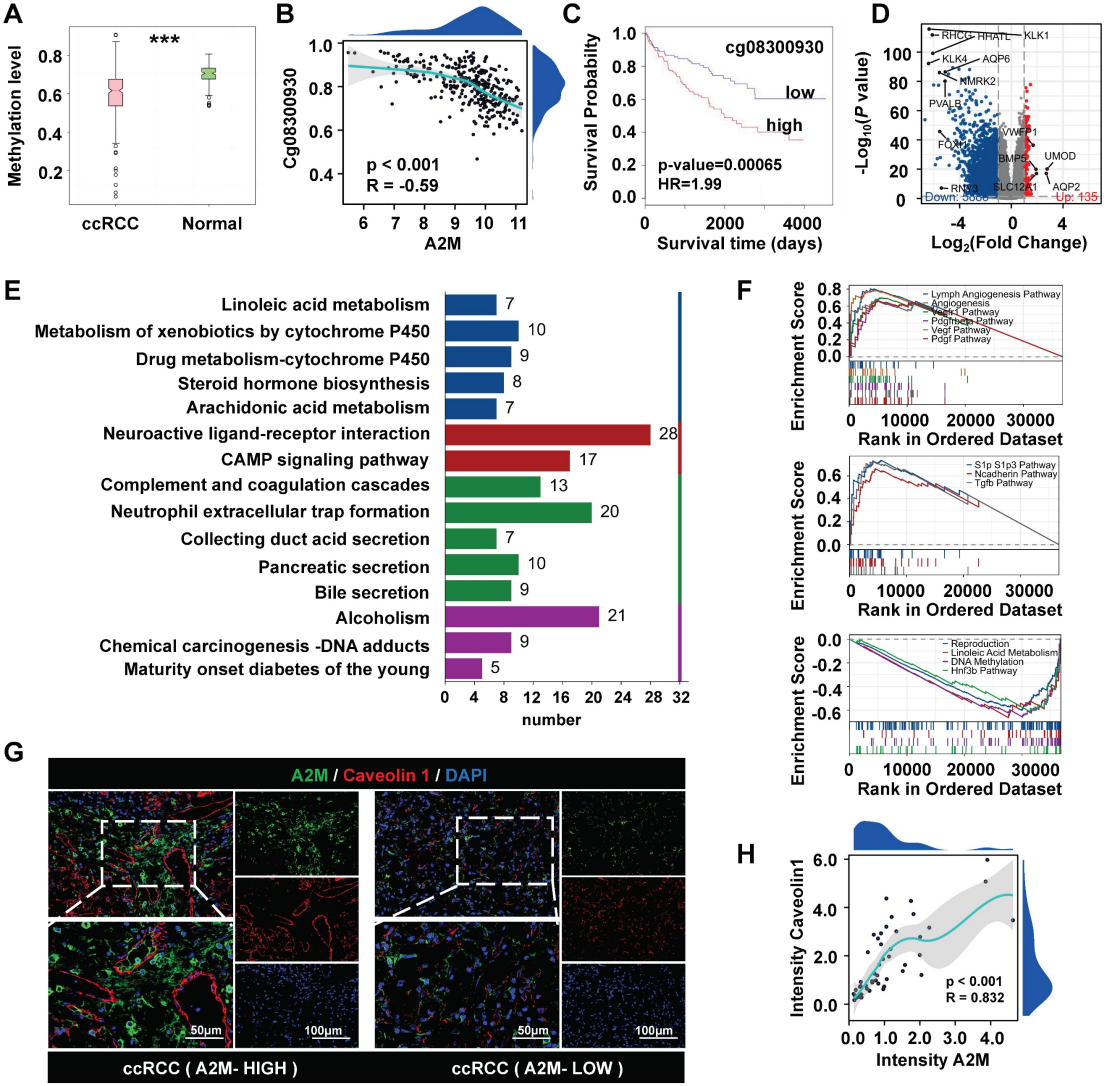
** A2M single-gene difference analysis. (A)** Detection of A2M methylation level in normal kidney and ccRCC; **(B)** Spearman correlation analysis of the relationship between the methylation level of CpG site Cg08300930 of A2M gene in ccRCC and the expression level of A2M (p < 0.05 indicated a statistically significant difference); **(C)** Kaplan-Meier survival curve analysis of the relationship between A2M in the Cg08300930 hypermethylated group and hypomethylated group and survival time of ccRCC patients; **(D)** volcano map of A2M single-gene difference analysis results in the TCGA-KIRC cohort (blue: downregulated DEGs; red: upregulated DEGs; gray: unaltered genes); the top 5 upregulated genes with the largest |log2FC| and the top 10 downregulated genes with the largest |log2FC| were labeled; **(E)** KEGG enrichment analysis of DEGs in A2M single-gene analysis in TCGA-KIRC cohort (p < 0.05, |log2FC| > 1.5); **(F)** GSEA enrichment analysis of DEGs in A2M single gene analysis in TCGA-KIRC cohort; **(G-H)** double immunofluorescence map of A2M and Caveolin1 in ccRCC tissues of clinical cases and their correlation analysis; *p < 0.05, compared to normal; **p < 0.01, compared to normal; ***p < 0.001, compared to normal.

Next, the role of A2M in the development and progression of ccRCC was explored. A2M methylation level in ccRCC was observed and biological enrichment analysis was performed. It was found that the methylation level of A2M in ccRCC was reduced compared with that in normal people (Figure 4A), and obvious hypomethylation was observed in the Cg08300930 site of A2M gene (Figure 4B). A lower methylation level of this site of the A2M gene indicated a longer survival time of ccRCC patients (HR = 1.99, p = 0.00065) (Figure 4C). Additionally, the TCGA-KIRC cohort was analyzed for A2M single gene differences, and a total of 6023 significantly differential genes were identified (adjusted p < 0.05 and |log2FC| > 1.5). According to the volcano map of DEGs, there were 5888 downregulated genes and 135 upregulated genes (Figure 4D). Next, these DEGs in the ccRCC group were subject to enrichment analysis. GO enrichment analysis results showed that these differential genes were related to various molecular functions, cell composition, and biological processes, such as regulation of hormone levels, anchored component of membrane, and passive transmembrane transporter activity (Figure S6A & Table S5). KEGG enrichment analysis results indicated that A2M single-gene differential genes were involved in multiple biological pathways, such as cell complement and coagulation cascade, pigment P450-mediated exogenous metabolism, neuroactive ligand-receptor interactions, neutrophil-extracellular trap formation, and CAMP signaling pathway (Figure 4E & Table S6). Moreover, according to GSEA enrichment analysis results, genes in the A2M high expression group of ccRCC patients were mainly positively correlated with lymphatic angiogenesis, angiogenesis, VEGF, and VEGFR signaling pathways, and negatively correlated with signaling pathways such as reproduction, DNA methylation, and linoleic acid metabolism (Figure 4F & Table S7&8). Subsequently, for verifying the close correlation between high A2M expression and angiogenesis in ccRCC patients, the co-immunofluorescence of A2M and vascular endothelial marker Caveolin 1 in tumor tissues from clinical ccRCC patients in the First Affiliated Hospital of Jinan University was detected using the immunofluorescence method. The results indicated that A2M expression was positively correlated with the expression of endothelial cells (R = 0.832, p < 0.001) (Figure 4G&H), suggesting that A2M may play an important role in tumor angiogenesis in ccRCC.

### 3.5 The role of A2M in the regulation of immune invasion in ccRCC

**Figure 5:**
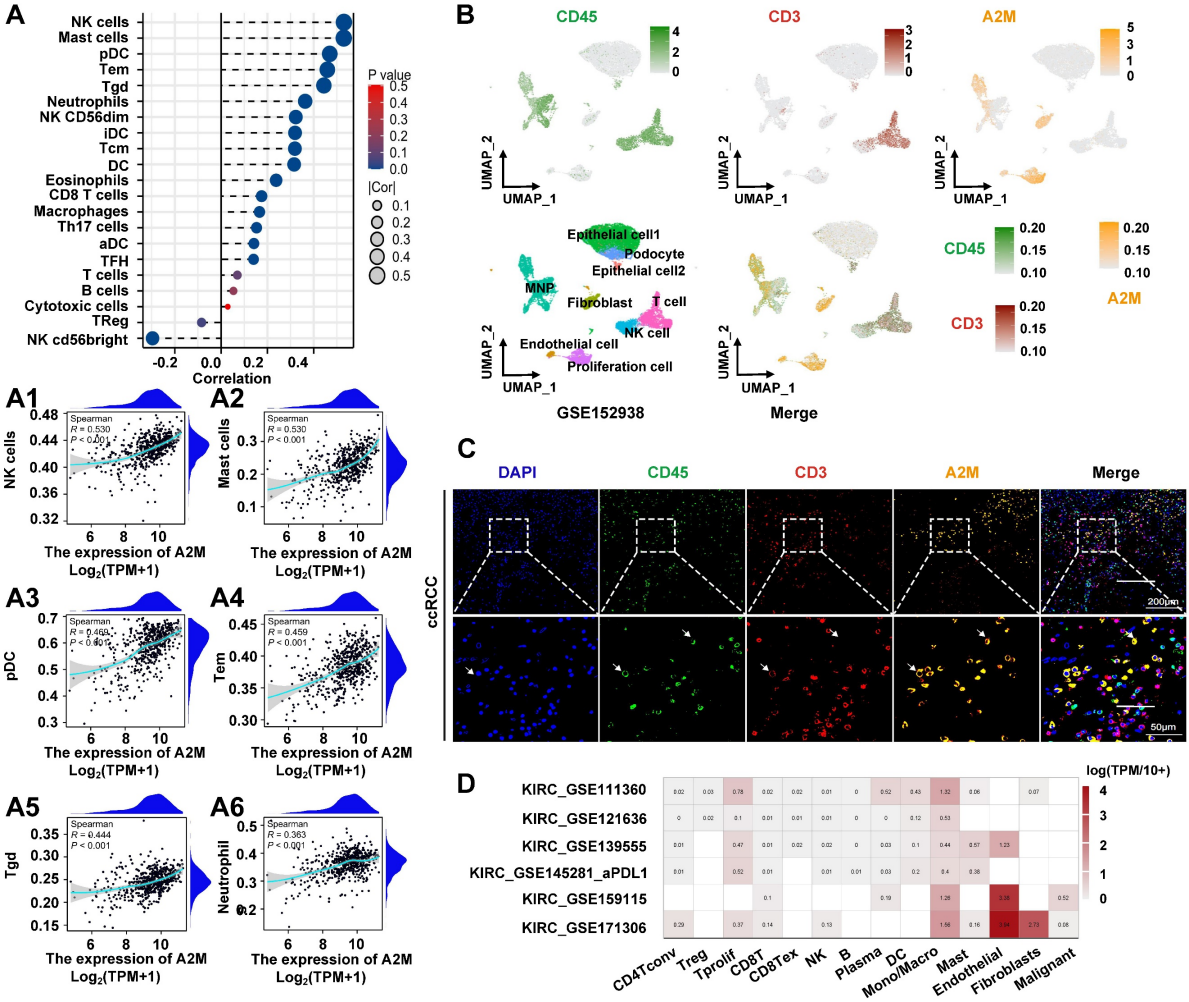
** The effect of A2M on immune infiltration in ccRCC. (A)** Correlation analysis of A2M expression and immune cell infiltration in ccRCC patients in the TCGA cohort; **(A1-A6)** correlation analysis between A2M expression and immune cells (NK cells, mast cells, pDC, Tem, Tgd, and neutrophils) (Top 6); **(B)** feature plot showing the cell clusters of CD45, CD3, and A2M in ccRCC single-cell data (GSE152938); **(C)** immunocolocalization of A2M, CD45, and CD3 in ccRCC tumor tissues of clinical patients; **(D)** validation of the distribution of A2M in various immune cells through external single-cell databases (KIRC-GSE111360, GSE121636, GSE139555, GSE145281_aPDL1, and GSE159115, and GSE171306).

The above results suggested that low A2M expression was associated with rapid tumor progression in ccRCC. However, the underlying mechanisms of A2M in RCC progression and the immune microenvironment remained unclear. Therefore, A2M single gene differences were first analyzed using the TCGA-KIRC, TCGA-KIRP, and TCGA-KICH databases, and enrichment analysis was conducted on the commonly differentially expressed genes (Co-DEGs) combined differential fold changes log2FC. Changes in A2M expression can cause the difference of 91 identical genes in ccRCC, pRCC, and chRCC, and these genes were enriched in multiple immune pathways (Figure S3 & Table S9). This suggested that A2M may play an important role in RCC immunomodulation. Therefore, immune infiltration analysis was first conducted on the TCGA-KIRC cohort and it was found that A2M was positively correlated with most of the immune cell types, with the most significant cell types including NK cells (R = 0.530, p < 0.001), mast cells (R = 0.530, p < 0.001), pDC (R = 0.469, p < 0.001), Tem (R = 0.459, p < 0.001), Tgd (R = 0.444, p < 0.001), Neutrophils (R = 0.363, p < 0.001), and macrophages (R = 0.166, p < 0.001) (Figure 5A&A1-A6). Moreover, A2M also showed a trend of positive association with intratumoral immune cell infiltration in rRCC and chRCC (Figure S4A-B), but the immune cell types with significant association were not the same as that in ccRCC. Next, the role of A2M in the immunity of ccRCC patients was further investigated. Specifically, the single-cell sequencing results were analyzed and it was found that A2M was primarily expressed in mononuclear phagocytes (MNPs), endothelial cells, fibroblasts, and proliferating cells in ccRCC dataset GSE152938; the single-cell expression position of a few leukocyte marker CD45 and T cell marker CD3 overlapped with the expression position of A2M, which suggested that A2M may be related to MNPs and T cells in immune cells, with a more close relation to MNP (Figure 5B). To further verify this conclusion, we used clinical ccRCC tumor tissues to co-locate A2M with leukocyte marker CD45 and T cell marker CD3 by immunofluorescence. The results showed that A2M was expressed in only a small part of CD45+CD3+ cells. This suggested that A2M may have a limited effect on T cells' immune function (Figure 5C). Next, the correlation between immune cell distribution and proportion and A2M expression level at the single-cell level was verified. Immune cell profiles in 6 external single-cell databases (KIRC_GSE111360, KIRC_GSE121636, KIRC_GSE139555, KIRC_GSE145281_aPDL1, KIRC_GSE159115, and KIRC_GSE171306) were analyzed, and high expression of A2M was also found in MNPs and Tprolif with high levels (Figure 5D). Additionally, in KIRP and KICH, A2M was also found highly expressed in mononuclear macrophage cell lines (Figure S5). Taken together, A2M can change the immune microenvironment of ccRCC by affecting MNPs and T cells.

**Figure 6:**
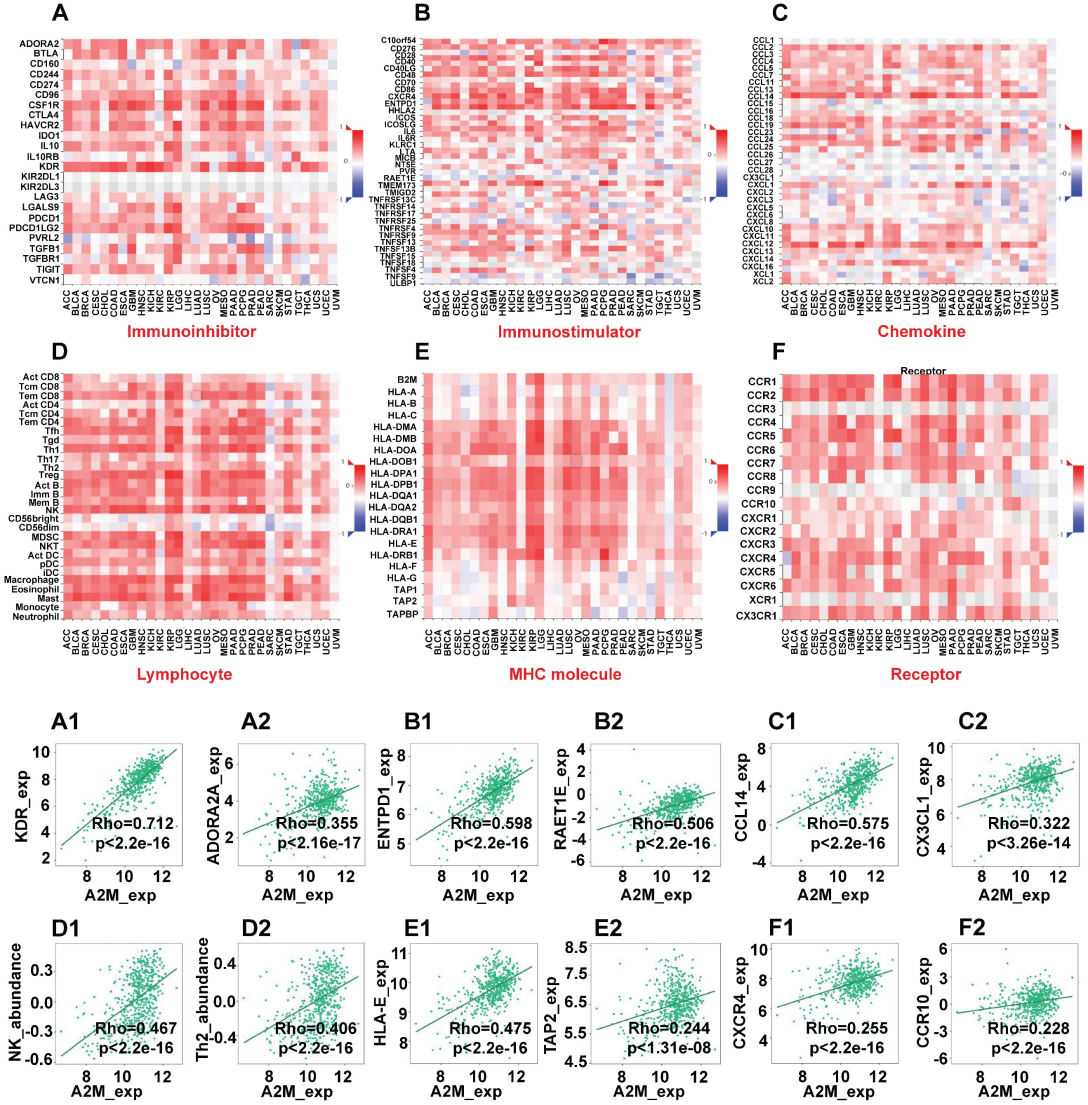
** A2M and immunotherapy response in ccRCC. (A-F)** The correlation between A2M expression and immunoinhibitors (A), immunostimulators (B), chemokines (C), lymphocytes (D), MHC molecules (E), and immune receptors (F) in pancarcinoma; **(A1-A2)** scatter plots of the top 2 immunoinhibitors positively correlated with A2M expression in ccRCC patients; **(B1-B2)** scatter plots of the top 2 immunostimulators positively correlated with A2M expression in ccRCC patients; **(C1-C2)** scatter plots of the top 2 chemokines positively correlated with A2M expression in ccRCC patients; **(D1-D2)** scatter plots of the top 2 lymphocytes positively correlated with A2M expression in ccRCC patients; **(E1-E2)** scatter plots of the top 2 MHC molecules positively correlated with A2M expression in ccRCC patients; **(F1-F2)** scatter plots of the top 2 immune receptors positively associated with A2M expression in ccRCC patients.

Next, the immunoinhibitors, immunostimulators, chemokines, lymphocytes, MHC molecules, and immune receptors that were significantly associated with A2M in the immunotherapy response of ccRCC patients were explored (Figure 6). The colors and their intensity on the bar scale denoted the nature of the correlation, with the darker shade of blue indicating more negative correlations (close to -1) and the darker shade of red indicating more positive correlations (closer to 1). It was found that the top two immunoinhibitors positively correlated with A2M in ccRCC were KDR (Cor = 0.712, p < 2.2e-16) and ADORA2A (Cor = 0.355, p < 2.16e-17) (Figure 6A1-A2). The top two immunostimulators positively correlated with A2M in ccRCC were ENTPD1 (Cor = 0.598, p < 2.2e-16) and RAET1E (Cor = 0.506, p < 2.2e-16) (Figure 6B1-B2). Additionally, CCL14 (Cor = 0.575, p < 2.2e-16) and CX3CL1 (Cor = 0.322, p < 3.26e-14) were top two chemokines positively correlated with A2M in ccRCC (Figure 6C1-C2). The top two lymphocytes positively correlated with A2M in ccRCC included NK (Cor = 0.467, p < 2.2e-16) and Th2 (Cor = 0.406, p < 2.2e-16) (Figure 6D1-D2). The top two MHC molecules positively correlated with A2M in ccRCC were HLA-E (Cor = 0.475, p < 2.2e-16) and TAP2 (Cor = 0.244, p < 1.31e-08) (Figure 6E1-E2). Finally, CXCR4 (Cor = 0.255, p < 2.2e-16) and CCR10 (Cor = 0.228, p < 2.2e-16) were top two immune receptors positively correlated with A2M in ccRCC (Figure 6F1-F2). Taken together, these results suggested that A2M may be involved in the regulation of tumor microenvironment by MNP and T cells in ccRCC, thus affecting immunotherapy response. Additionally, it was also found through the DrugBank database that some clinical drugs can alter the mRNA expression level of A2M. Antimony compounds and immunoinhibitor cyclosporine can reduce A2M mRNA expression, while organoarsenic drugs DARVIAS (darinaparsin), dasatinib, dexamethasone, isotretinoin, selenium, and silicon dioxide can increase A2M mRNA expression (Table 2).

### 3.6 Analysis of A2M closely related genes and their variation landscape in ccRCC patients

**Figure 7:**
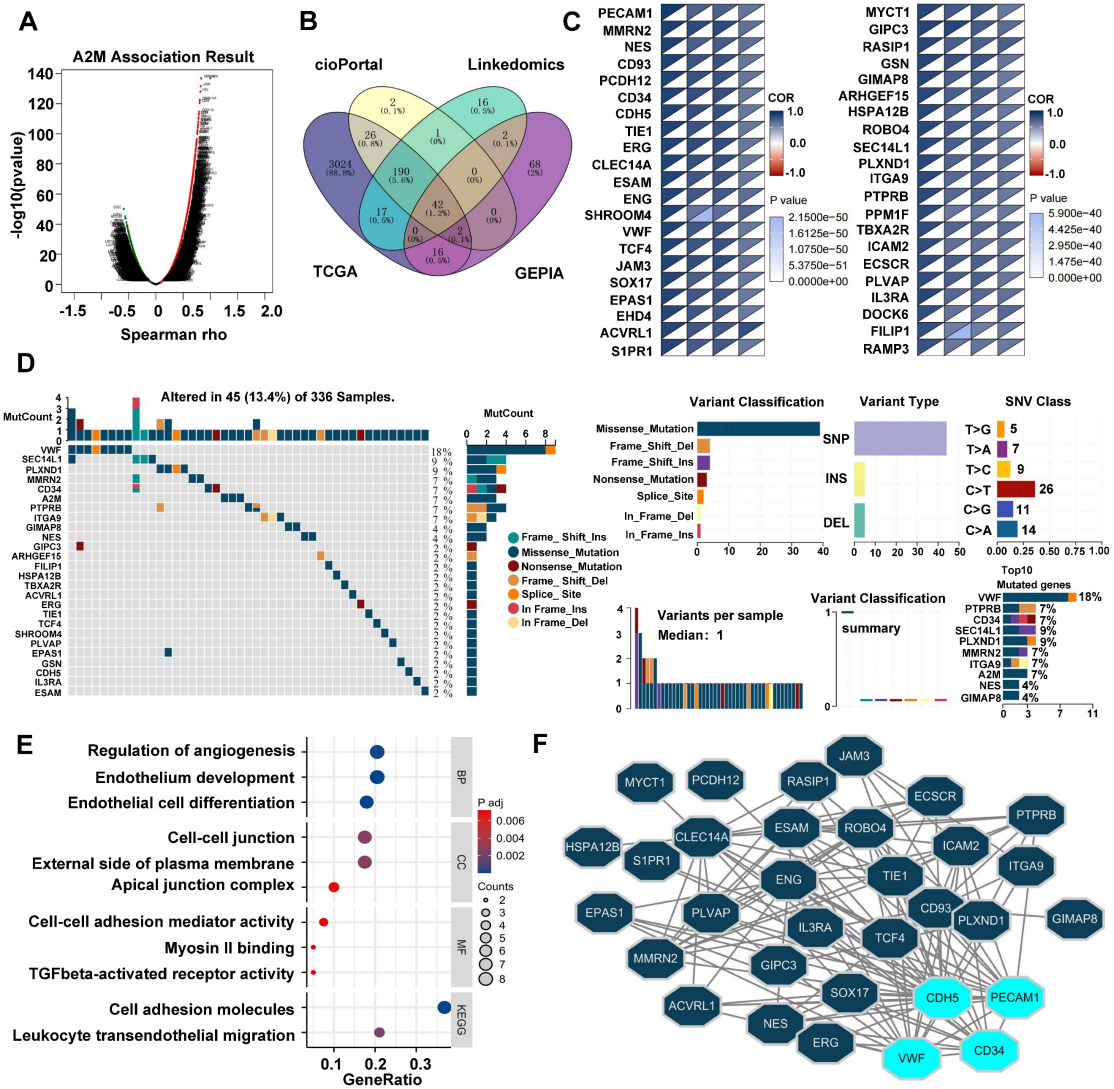
** Analysis of A2M-related differential genes and tumor mutations in ccRCC. (A)** Volcano map of A2M single gene correlation analysis; **(B)** Venn diagram of the intersection of A2M-related genes in four different databases (TCGA, GEPIA, cBioPortal, and LinkedOmics) (Cor-spearman ≥ 0.6); **(C)** the correlation degree between 42 co-related genes and A2M in four databases; **(D)** waterfall plot of A2M-related genes in TCGA cohort; mutation summary map showing mutation classification, mutation distribution of single nucleotide variants (SNV) category, and top 10 mutated genes; **(E)** GO and KEGG enrichment analysis of co-related genes; **(F)** the PPI network of A2M co-related genes via Cytoscape visualization; the top 4 significantly interacting genes with the highest PPI degree (degree ≥ 15) were selected.

The potential role of A2M in tumorigenesis and tumor progression was further explored. Spearson correlation analysis of A2M genes was first conducted using the LinkedOmics database. According to the results, there were 9187 genes positively correlated with A2M and 10924 genes negatively correlated with A2M (Figure 7A). After intersecting A2M-related genes in TCGA, GEPIA, cBioPortal, and LinkedOmics databases (cor-spearman ≥ 0.6), 42 A2M co-related genes (1.2%) were identified, including PECAM, MMRN2, NES, CD93, etc. (Figure 7B-C). Additionally, the somatic variation of 42 A2M co-related genes in ccRCC patients was analyzed, and the results showed that about 13.4% (45/336) of ccRCC patients had mutations in A2M co-related genes. Among the top 10 mutated genes, Von Willebrand factor (VWF) (18%) had the highest mutation frequency, and the other 9 genes had a mutation frequency of 4%-9%; missense mutations accounted for the vast majority of mutation types (Figure 7D). Further GO and KEGG enrichment analyses of A2M co-related genes showed that A2M co-related genes were mainly involved in regulating various biological processes such as angiogenesis, endothelial cell development and differentiation, and cell adhesion (Figure 7E& Table S10). As indicated by the protein-protein interaction (PPI) network analysis results of 42 A2M co-related genes, CD34, PECAM1, CDH5, and VWF had tight interprotein interactions (Figure 7F). The correlation coefficient diagram showed the internal correlations between CD34, PECAM1, CDH5, VWF, and A2M genes, among which A2M and PECAM1 had the strongest correlation (R = 0.90) (Figure S6B). According to Kaplan-Meier curve analysis results, high CD34, PECAM1, CDH5, and VWF mRNA expressions were associated with longer OS in ccRCC patients in GEPIA (p < 0.001) (Figure S6C). These findings further suggested that A2M in ccRCC played an important role in tumor angiogenesis.

### 3.7 Screening of prognostic characteristic variables in ccRCC patients and construction of the A2M-GPI prognostic model

**Figure 8:**
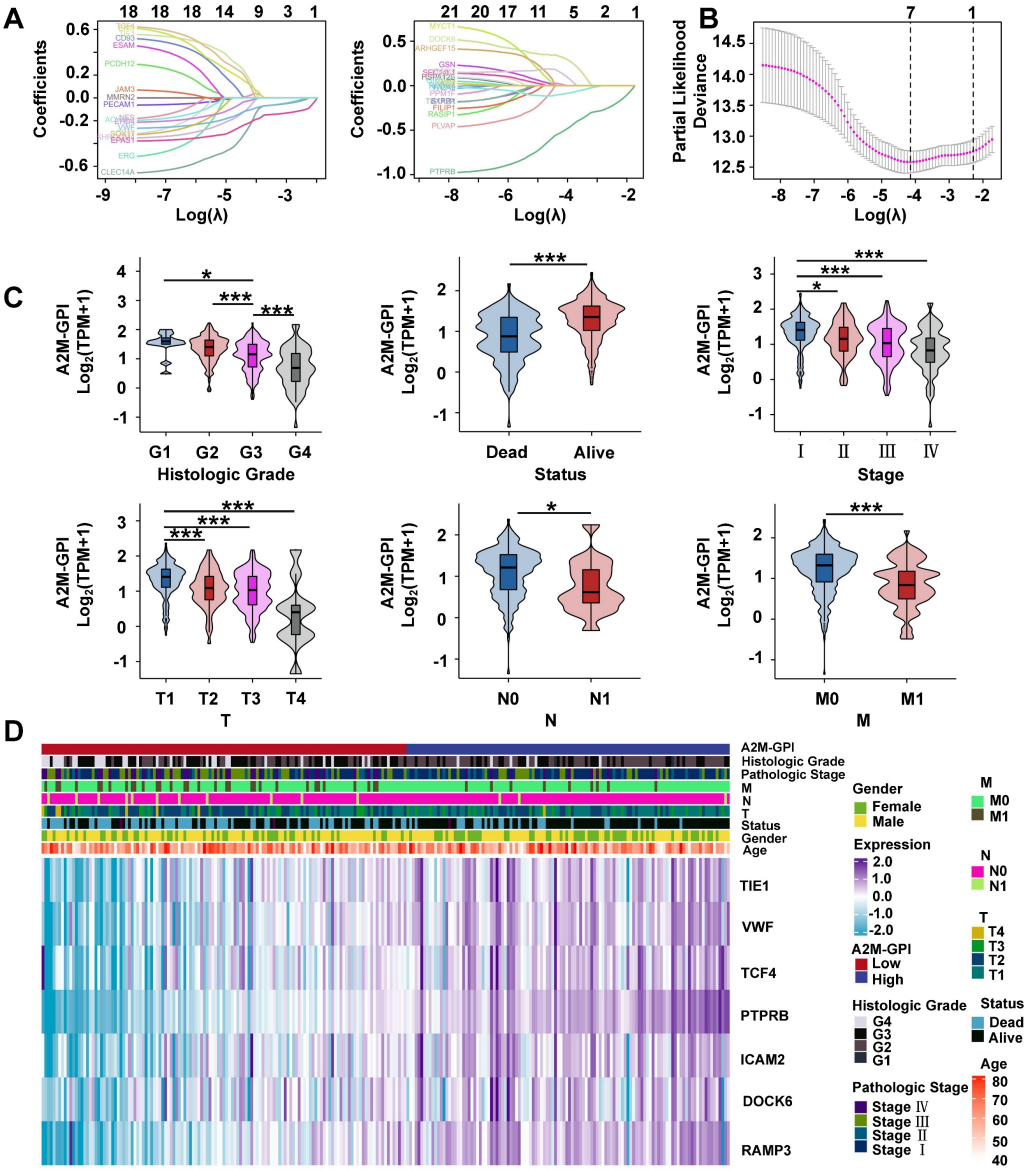
** Screening of prognostic characteristic variables in ccRCC patients. (A)** Selection of 7 model genes by machine learning; **(B)** 10-fold cross-validation of model characteristic variables; **(C)** Violin diagram showing the relationship between A2M-GPI and tissue grade, survival status, pathological stage, and TNM stage of tumor;** (D)** heat map of 7 model genes and clinical features. *p < 0.05; **p < 0.01; ***p < 0.001.

Clinical survival information of ccRCC patients was collected and analyzed. Univariate Cox analysis was performed on 42 A2M co-related genes to screen survival-related genes (p < 0.05). Through LASSO-Cox regression analysis, 7 genes were identified [TIE1, VWF, transcription factor 4 (TCF4), protein tyrosine phosphatase receptor type B (PTPRB), intercellular adhesion molecule 2 (ICAM2), dedicator of cytokinesis 6 gene (DOCK6), and receptor activity modifying protein-3 (RAMP3)] and the A2M-GPI prognostic model was constructed (Figure 8A-B). The A2M-GPI score of each patient was obtained by the following formula: A2M-GPI = (-0.06057755*TIE1 exp.) + (0.00416184*VWF exp.) + (-0.22620967*TCF4 exp.) + (0.62309290*PTPRB exp.) + (-0.06403383*ICAM2 exp.) + (-0.19163895*DOCK6 exp.) + (0.08291625* RAMP3 exp.). Based on the median A2M-GPI value calculated by the above formula, patients with clinical information in the TCGA cohort were allocated into the high A2M-GPI and low A2M-GPI group and served as a training set. A2M-GPI was significantly correlated with various clinical features. For example, in histologic grade (G1-G4), pathologic stage (I-IV), T (T1-T4), N (N0-N1), and M (M0-M1), the A2M-GPI value was decreased with the increase of histologic grade. The level of A2M-GPI in surviving patients was markedly higher than that in dead patients (Figure 8C-D).

### 3.8 Internal training and external validation of A2M-GPI predictive models

**Figure 9:**
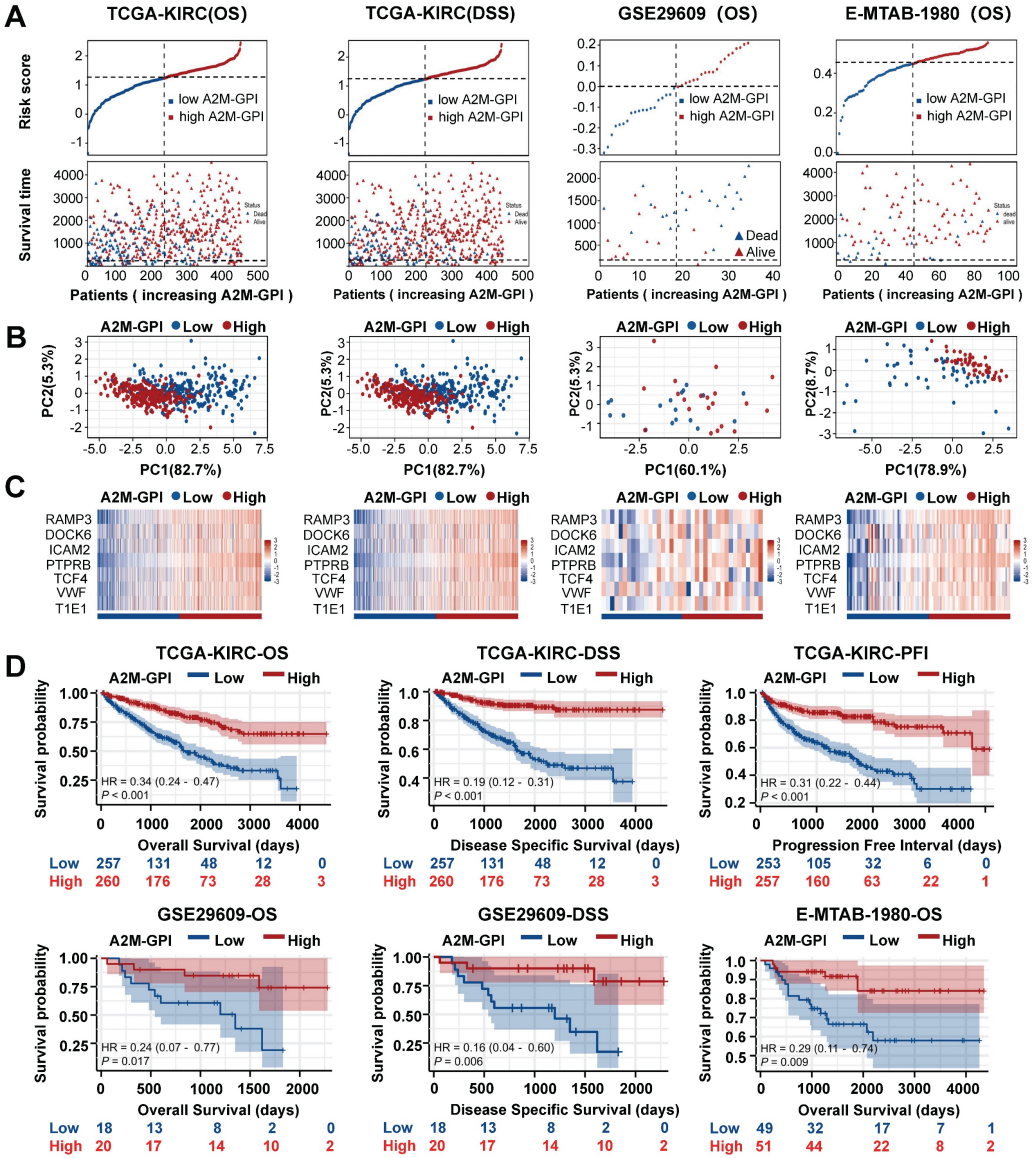
** Internal training and external verification of gene signature prognostic prediction model. (A)** Distribution of A2M-GPI adjusted for survival state and time in TCGA, E-MTAB-1980, and GSE29609 cohorts; **(B)** PCA diagram of A2M-GPI in TCGA-KIRC (OS), TCGA-KIRC (DSS), E-MTAB-1980 and GSE29609 cohorts; **(C)** the expression of each gene component in the high or low A2M-GPI risk group;** (D)** OS, DSS, and PFI in patients with high A2M-GPI and low A2M-GPI in the TCGA-KIRC cohort; OS and DSS in patients with high A2M-GPI and low A2M-GPI in the GSE29609 cohort; OS in patients with high A2M-GPI and low A2M-GPI in the E-MTAB-1980 cohort.

Next, the survival rates of ccRCC patients with different A2M-GPI values were compared. In short, 517 ccRCC patients in the TCGA-KIRC (OS) cohort with OS > 60 days were allocated into the high A2M-GPI risk group and low A2M-GPI risk group based on A2M-GPI score combined survival status, and this group was selected as the training dataset. First, the OS of ccRCC patients with different A2M-GPI scores was compared. The results showed that patients with high A2M-GPI had better OS than those with low A2M-GPI (Figure 9A). PCA results revealed that the clustering effect based on A2M-GPI was satisfactory (Figure 9B). Moreover, as shown by the heat map, genes of each component in the group with high A2M-GPI also showed higher expression levels (Figure 9C). Additionally, survival data showed that ccRCC patients with high A2M-GPI scores had higher OS (p < 0.001) (Figure 9D).

Meanwhile, TCGA-KIRC (DSS), E-MTAB-1980, and GSE29609 were used as model external validation cohorts. The results showed that there were significant differences in the number of survival and death status distributions between the high A2M-GPI risk group and low A2M-GPI risk group (Figure 9A), and the clustering was satisfactory. ccRCC patients with high A2M-GPI scores had a better prognosis both in DSS and PFI (Figure 9D).

### 3.9 Establishment, evaluation, and clinical application of the nomogram model

**Figure 10:**
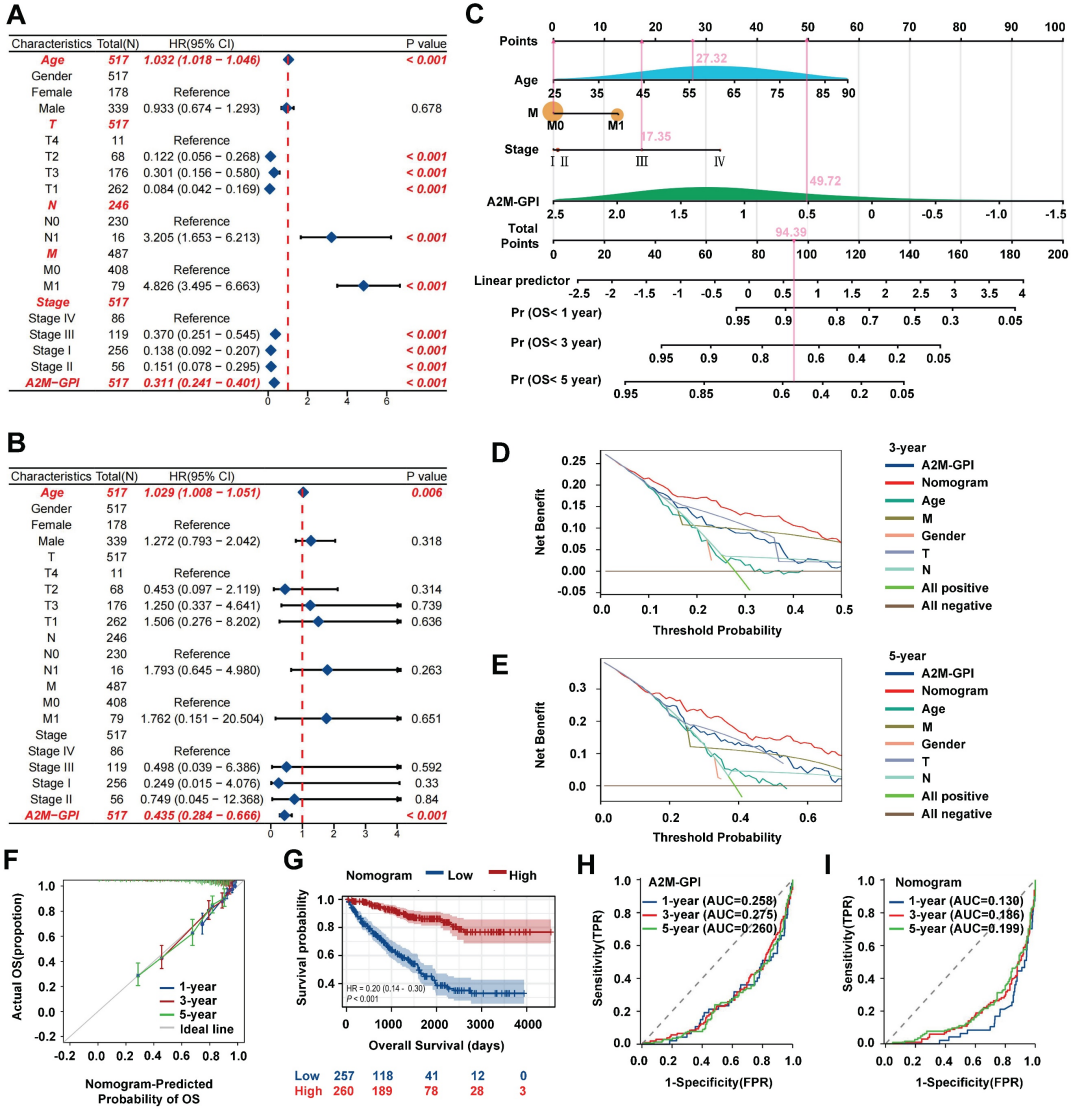
** Establishment, evaluation, and clinical application of the nomogram survival diagram. (A)** Univariate Cox analysis of A2M-GPI and clinicopathological features in the TCGA-KIRC cohort; **(B)** multivariate Cox analysis of A2M-GPI and clinicopathological features in the TCGA-KIRC cohort;** (C)** a nomogram for predicting the prognosis of ccRCC patients; **(D)** DCA for nomogram prediction of 3-year OS; **(E)** DCA for nomogram prediction of 5-year OS; **(F)** prognostic calibration diagram showing the 1-, 3-, and 5-year OS probabilities of ccRCC patients in the TCGA cohort; **(G)** Kaplan-Meier analysis of ccRCC patients in the low nomogram and high nomogram groups based on the nomogram score; **(H)** ROC analysis of the A2M-GPI model for 1-, 3-, and 5-year prognosis of ccRCC in the TCGA cohort; **(I)** ROC analysis of the nomogram model for 1-, 3-, and 5-year prognosis of ccRCC in the TCGA cohort.

A2M-GPI showed good clustering and prognostic effects in internal training and external verification. Subsequently, whether A2M-GPI could be an independent prognostic factor for ccRCC patients was further determined. Univariate and multivariate Cox regression analysis was performed on A2M-GPI and other clinical variables (Figure 10A-B). According to univariate Cox regression analysis results, age, T, N, M, pathologic stage, and A2M-GPI of ccRCC patients could significantly affect the prognosis of patients. Age (HR = 1.032, 95%CI: 1.018-1.046, p < 0.001), lymphatic metastasis (HR = 3.205, 95%CI: 1.653-6.213, p < 0.001), and distant metastases (HR = 4.826, 95%CI: 3.495-6.663, p < 0.001) were considered risk factors, whereas A2M-GPI was considered a protective factor (HR = 0.311, 95%CI: 0.241-0.401, p < 0.001) (Figure 10A). Multivariate Cox regression analysis results indicated that A2M-GPI could be used as an independent prognostic factor in ccRCC patients (HR = 0.435, 95%CI: 0.284-0.666, p < 0.001). Additionally, age was also an independent prognostic factor (HR = 1.029, 95%CI: 1.008-1.051, p = 0.006) (Figure 10B). Considering data accessibility and clinical applicability, 4 clinical characteristic variables (age, stage, presence of distant metastasis, and A2M-GPI) were used to construct the nomogram model. Multivariate Cox and stepwise regression analysis were used to construct a nomogram survival prognosis model based on the TCGA-KIRC cohort and to show the 1-, 3-, and 5-year OS probabilities of patients (Figure 10C). The C-index of the model was 0.786 (95%CI: 0.768-0.804). In the 3-year and 5-year decision curve analysis (DCA), it can be seen that the constructed nomogram model had better benefit, specificity, and sensitivity than any other characteristic variable and model (Figure 10D-E). The prognostic calibration curves showed a good fit between the OS probability predicted by the nomogram model and actual survival at 1, 3, and 5 years (Figure 10F). Kaplan-Meier survival analysis results showed that there were significant differences in OS between high and low nomogram scores for ccRCC patients (p < 0.001), with patients with high nomogram scores having higher OS rates (Figure 10G). Next, the diagnostic value of the component genes, A2M-GPI model, and nomogram model at 1, 3, and 5 years was analyzed through ROC curves. The AUC indicated that the nomogram model had higher diagnostic value and accuracy in predicting prognostic survival of ccRCC patients (Figure 10H-I and Figure S6D). Moreover, a web site (https://A2Mgpinomogram.shinyapps.io/ccRCC_prognosis_prediction/) used by the users was constructed to facilitate the usage of our prediction model by the clinical doctors. The input data (age, stage, TNM stage, and A2M-GPI) were pre-processed, and then the 1-, 3-, and 5-year survival probability and survival maps were automatically exported.

## Discussion

A2M belongs to the I39 MEROPS family, which has 7 members in humans and 2 members in mice. A2M is also involved in multiple functional regulation in the body. A2M can improve immunity, enhance antigen uptake, processing, and presentation of antigen-presenting cells 17-19. Therefore, A2M can be used as a biomarker for the diagnosis and prognosis of many diseases. Although several studies have explored A2M expression and function in specific tumors, its broader role—particularly its prognostic implications—has not been systematically investigated. In this study, we provided the first comprehensive analysis of the expression level and prognostic value of A2M between tumor and normal tissues. Our study showed that the mRNA expression of A2M was downregulated in most tumors. Existing evidence has documented that A2M expression is significantly upregulated in a few tumors such as KIRC, DLBC, and GBM. Additionally, we assessed its prognostic value in multiple tumors and found significant correlations in SARC, SKCM, and KIRC, suggesting distinct roles in tumor biology.

Recently, Cheng et al. 20 have identified 5 complement-related genes (A2M, APOBEC3G, COL4A2, DOCK4, and NOTCH4) through a comprehensive analysis of mRNA expression data in the database of the International Cancer Genome Consortium, and established a risk score model to predict the prognosis of ccRCC. However, little is known about the expression of A2M in ccRCC tumors and its prognostic and therapeutic value. In ccRCC specifically, we conducted a detailed analysis of A2M's expression profile, diagnostic/prognostic relevance, methylation status, somatic mutations, immune infiltration, and functional pathways for the first time—identifying it as a strong prognostic biomarker.

Our study suggested that A2M is a potential novel prognostic marker in ccRCC. Over the past decade, the treatment of RCC has overgrown to targeted therapies targeting specific targets, such as VEGF, PDGF, and related receptors 21. A2M-related differential genes in ccRCC were screened and enriched through GO, KEGG, and GSEA analyses. These differential genes were enriched in the regulation of hormone levels and cell complement and coagulation cascade. Interestingly, according to GSEA enrichment results, high expression of A2M in ccRCC patients was positively correlated with lymphatic angiogenesis, angiogenesis, VEGF, VEGFR, and other signaling pathways. This suggested that these findings were closely related to the important role of A2M in angiogenesis. It has been shown that A2M binds with various important vascular genetic factors [such as bFGF, VEGF, and placental growth factor (PlGF)] to inactivate the binding factors 22. Additionally, A2M can also regulate uterine vascularization and remodeling during pregnancy 23. Subsequently, double immunofluorescence labeling of A2M and vascular marker Cavolin1 was further performed to explore whether A2M expression in ccRCC was closely related to the number of vascularization. Although there was no colocalization between the two, it was found that Cavolin1 expression was increased around the region with high A2M expression, which may be attributed to the complexity of tumor vessels 24.

In recent years, the introduction of ICI therapy has effectively improved the prognosis of RCC patients 25. There are various immune cells in the tumor microenvironment, which gather in the tumor tissue and play a certain anti-tumor role. The body recruits various immune cells (such as mononuclear macrophages and T cells) to eliminate tumor cells 26, 27. This study revealed that A2M played an important role in tumor immune regulation and was closely related to NK cells, MNPs, and T cells. It has been evidenced that NK cell infiltration has a protective effect and has prognostic value in renal, colorectal, and lung cancer 28-30. Additionally, Yulin Deng et al. have found that the ccRCC group with a low risk of somatic cell mutation has a higher abundance of activated NK cells 31. However, the specific mechanism of A2M in regulating the activity of NK cells in ccRCC to inhibit tumor growth remains to be further studied. As an important component of the immune tumor microenvironment, MNP is specifically involved in cancer-specific T-cell-mediated killing of tumor cells, which provides new possibilities for cancer immunotherapy 32. This study found that A2M was closely related to MNP. Consistently, studies have shown that A2M induces changes in internal conformation when stimulated, thus stimulating the receptor-mediated endocytosis of the MNP system 33, 34. Therefore, it's hypothesized that the formation of the MNP immune microenvironment was also involved in the anti-tumor effect of A2M, and A2M may also act as an indirect immune target regulated by MNP. It's well-established that infiltrating T cells play an important role in shaping the anti-tumor immune response; CD8+ T cells, in particular, are one of the major immune cell types responsible for tumor cell killing 35. Accumulating reports have shown that the number and density of tumor-infiltrating CD8+ T cells can improve the survival rate of cancer patients 36, 37. In the complex tumor microenvironment, constant antigen exposure of T cells can result in dysfunction, and dysfunctional T cells have reduced proliferation ability, presenting a low-functioning state of "T cell exhaustion." Therapeutic reactivation of tumor-specific T cells has yielded promising results in cancer patients 38. A2M has the function of antigen delivery, which can enhance the response of T lymphocytes 19, participating in the immune microenvironment of tumors.

Subsequently, the role of A2M in ccRCC was further investigated. In short, the interaction between A2M genes and neighboring genes with frequent mutations was explored to identify the potential causes of A2M changes. In the network, 42 genes in four databases that shared a close positive association with A2M were identified, and the mutation of these 42 genes was observed. The results suggested that VWF had the highest mutation frequency (18%), and missense mutations accounted for the vast majority of mutation types. Consistently, it has been evidenced that elevated VWF levels in cancer patients may not only lead to cancer-related clotting disorders but can also mediate cancer progression and metastasis. Endothelium-secreted VWF polymers contribute to tumor cell adhesion and transendothelial migration, which is critical for tumor transmission 39. VWF mutations cause functional deficits, often resulting in blood disorders and rare tumor formation 40. This further confirmed that the role of A2M in ccRCC was closely related to tumor angiogenesis. Additionally, it was also found that among 42 A2M co-related genes, CD34, PECAM1, CDH5, and VWF had tight protein interactions, which further proved that the role of A2M in ccRCC was closely related to angiogenesis.

In addition to biomarkers, the emergence and development of prognostic models in recent years have also played a key role in the diagnosis and treatment of RCC, which compensates for deficiencies in targeting individual gene alterations. Through the intersection of A2M-related genes in multiple ccRCC databases, 42 A2M co-related genes were identified, and then subject to univariate Cox analysis. Next, 7 genes (TIE1, VWF, TCF4, PTPRB, ICAM2, DOCK6, and RAMP3) were screened to construct the prognostic model. These 7 genes have been reported to be involved in various biological processes (such as angiogenesis, transcriptional regulation, signaling, cell adhesion). In short, it has been evidenced that TIE1 is involved in tumor angiogenesis and inflammatory vascular remodeling 41. TIE1 mRNA expression is elevated in RCC, and TIE1 receptor and its ligands may play a role in RCC angiogenesis 42. The transcription factor TCF4 has also been shown to be associated with RCC invasiveness 43. VWF can promote pro-inflammatory signaling, regulate angiogenesis and vascular permeability, and thus promote tumor cell growth and vascular metastasis 39. In ccRCC, VWF expression level in elderly patients aged over 65 years is markedly higher than that in younger (< 65 years) patients 44. PTPRB is a transmembrane protein associated with endothelial cell adhesion, which can inhibit tumor cell proliferation and invasiveness 45. Unfortunately, PTPRB has been poorly studied in RCCS. ICAM2 is a transmembrane glycoprotein, and erythroblast transformation-specific-related gene (ERG)-induced ICAM2 can inhibit tumor proliferation and metastasis by enhancing ubiquitination and degradation of radixin (RDX) 46. DOCK6 is one of the novel age-related biomarkers for identifying and validating thyroid cancer, which can predict prognosis and immunotherapy 47. RAMP3 is one of three members of the RAMPs family, with RAMP3 deletion leading to inhibition of tumor proliferation and metastasis 48. To date, no study has evaluated the prognostic value of these genes in ccRCC patients. To compare the prognostic efficacy of our A2M-based nomogram model (C-index = 0.786) with existing ccRCC models, we integrated previous studies that utilized features with distinct biological significance, such as metabolism (C-index = 0.774) 49, cuproptosis (C-index = 0.77) 50, ferroptosis (C-index = 0.771) 51, glycolysis (C-index = 0.781) 52, m6A and m5C (C-index = 0.737) 53, potassium channels (C-index = 0.76) 54, tumor microenvironment (C-index = 0.744) 55, and immune features (C-index = 0.786) 56. Notably, our nomogram model demonstrated superior C-index performance compared to nearly all existing models in the TCGA-ccRCC dataset. In summary, these findings confirm that the A2M-centered nomogram model is a more effective prognostic tool for ccRCC. In addition, for the first time, this prognostic model based on A2M was established on a public website.

Over the past decade, the introduction of novel therapies (such as immune checkpoint inhibitors) has significantly advanced the clinical progress in the treatment of ccRCC. However, despite the remarkable efficacy of these novel therapies in some patients, a considerable proportion of patients still face challenges related to primary or acquired resistance. Therefore, there is an urgent need in clinical practice to explore new therapeutic strategies to further optimize treatment outcomes. The role of A2M in the treatment of ccRCC is still unclear. However, several studies have indicated that A2M demonstrates significant potential in tumor immunotherapy and targeted therapy 57. A2M plays a significant role in immunotherapy by enhancing antigen presentation, thereby improving the immune system's capacity to recognize and eliminate tumor cells 58. Additionally, A2M can also regulate the tumor microenvironment and affect the efficacy of immunotherapy. It has been shown that in patients with advanced prostate cancer, lower A2M levels are associated with lower levels of cytokines (such as IL-6 and TGF-Beta) 59. Both of them can affect the efficacy of PD-L1 cancer immunotherapy 60. Moreover, A2M can directly interact with multiple proteins of the complement system and influence their activation and regulation, thereby affecting the efficacy of immunotherapy. Chronic lymphocytic leukemia (CLL) is a hematologic malignancy characterized by the clonal proliferation of mature B lymphocytes. In CLL patients, A2M impacts the complement-dependent mechanisms activated by immunotherapeutic drugs through its involvement in the classical pathway of the complement system, consequently reducing the effectiveness of immunotherapy 61. Additionally, A2M can serve as a drug carrier to deliver immunotherapy drugs accurately to the tumor site, improving the therapeutic effect. CpG oligodeoxynucleotides (ODNs) represent a promising class of immunotherapeutic agents that activate the innate immune system through the Toll-like receptor 9 (TLR9) signaling pathway and exert anti-tumor effects by reversing the immunosuppressive tumor microenvironment. The combined use of CpG ODNs with A2M can markedly enhance immunostimulatory properties and elicit a more potent cytokine response. Furthermore, A2M-bound CpG ODNs gain nuclease protection that prevents their degradation, ultimately leading to improved efficacy of immunotherapy 62. Taken together, these studies provide critical references for further exploration of A2M's potential applications in therapeutic strategies.

## 5. Strengths and limitations

Despite the good performance of our model in both the training and validation cohorts, there are some limitations. First of all, most of the A2M expression and prognosis data came from existing data of different database cohorts, which is a retrospective study. Although some results have been revalidated, there are still inevitably varying degrees of bias. Second, the data in the online database are constantly updated, which may affect the final study results. Third, there is a lack of research on the mechanism by which A2M affects ccRCC. Fourth, the established model was only validated in the ccRCC public database, and validation through phase 3 randomized controlled trials is lacking. In the future, more high-quality, large samples, multi-center randomized controlled trials with adequate follow-up would be included for further verification, which is the direction of our efforts.

## 6. Conclusions

Therefore, this study aimed to analyze the expression of A2M in ccRCC and identify the relationship between A2M and the prognostic outcomes of ccRCC patients. Moreover, a new prognostic A2M gene-associated prognostic index (A2M-GPI) was established to predict the therapeutic effects of A2M on the intervention and prognosis of ccRCC. In conclusion, our study identified the heterogeneity of A2M in RCC patients and assessed the value of A2M in the clinical prognosis of ccRCC. Additionally, the establishment of the new model may help evaluate ccRCC patients and select appropriate treatment options. Additionally, the related prognostic model and the model application practice public website (https://A2Mgpinomogram.shinyapps.io/ccRCC_prognosis_prediction/) were established, which is of great significance for clinical prognosis assessment of ccRCC patients.

## Supplementary Material

Supplementary figures and tables.

## Figures and Tables

**Table 1:** Clinical baseline data table of patients in the TCGA ccRCC cohort.

Characteristic	Low expression of A2M	High expression of A2M	p
n	270	271	
T stage, n (%)			< 0.001
T1	116 (43%)	163 (60.1%)	
T2	43 (15.9%)	28 (10.3%)	
T3	103 (38.1%)	77 (28.4%)	
T4	8 (3%)	3 (1.1%)	
N stage, n (%)			0.383
N0	124 (92.5%)	118 (95.2%)	
N1	10 (7.5%)	6 (4.8%)	
N Unknown	136 (NA)	147 (NA)	
M stage, n (%)			< 0.001
M0	198 (78.9%)	231 (89.9%)	
M1	53 (21.1%)	26 (10.1%)	
M Unknown	19 (NA)	14 (NA)	
Pathologic stage, n (%)			< 0.001
Stage I	113 (42%)	160 (59.5%)	
Stage II	33 (12.3%)	26 (9.7%)	
Stage III	68 (25.3%)	55 (20.4%)	
Stage IV	55 (20.4%)	28 (10.4%)	
Stage Unknown	1 (NA)	2 (NA)	
Histologic grade, n (%)			< 0.001
G1	2 (0.8%)	12 (4.5%)	
G2	92 (34.8%)	144 (53.5%)	
G3	121 (45.8%)	85 (32%)	
G4	49 (18.6%)	27 (10%)	
Grade Unknown	6 (NA)	3(NA)	
OS event, n (%)			< 0.001
Alive	155 (57.4%)	211 (77.9%)	
Dead	115 (42.6%)	60 (22.1%)	
Age, n (%)			0.212
<= 60	127 (47%)	142 (52.4%)	
> 60	143 (53%)	129 (47.6%)	

**Table 2:** Drugs targeting A2M in the Drugbank.

Drug	Change	Interaction	References (PubMed ID)
Antimony	downregulated	Antimony results in decreased expression of A2M mRNA	17547211
Cyclosporine	downregulated	Cyclosporine results in decreased expression of A2M mRNA	20106945 25562108
Darinaparsin	upregulated	Darinaparsin results in increased expression of A2M mRNA	22535156
Dasatinib	upregulated	Dasatinib results in increased expression of A2M mRNA	20579391
Dexamethasone	upregulated	Dexamethasone results in increased expression of A2M mRNA	25047013
Isotretinoin	upregulated	Isotretinoin results in increased expression of A2M mRNA	20436886
Selenium	upregulated	Selenium results in increased expression of A2M mRNA	18997278
Silicon dioxide	upregulated	Silicon Dioxide analog results in increased expression of A2M mRNA	23806026
