# Supplementary material for: Alpha-2-Macroglobulin and Signature Genes: Predictive Biomarkers for Prognosis and Immunotherapy in Clear Cell Renal Cell Carcinoma
Source: J Cancer. 2025 Jul 10;16(10):3141–62. doi: 10.7150/jca.113242 (PMC12305615; doi:10.7150/jca.113242)
Supplement: Supplementary file 1 — Supplementary figures and tables. [file jcav16p3141s1.pdf]

Figures S1- S6

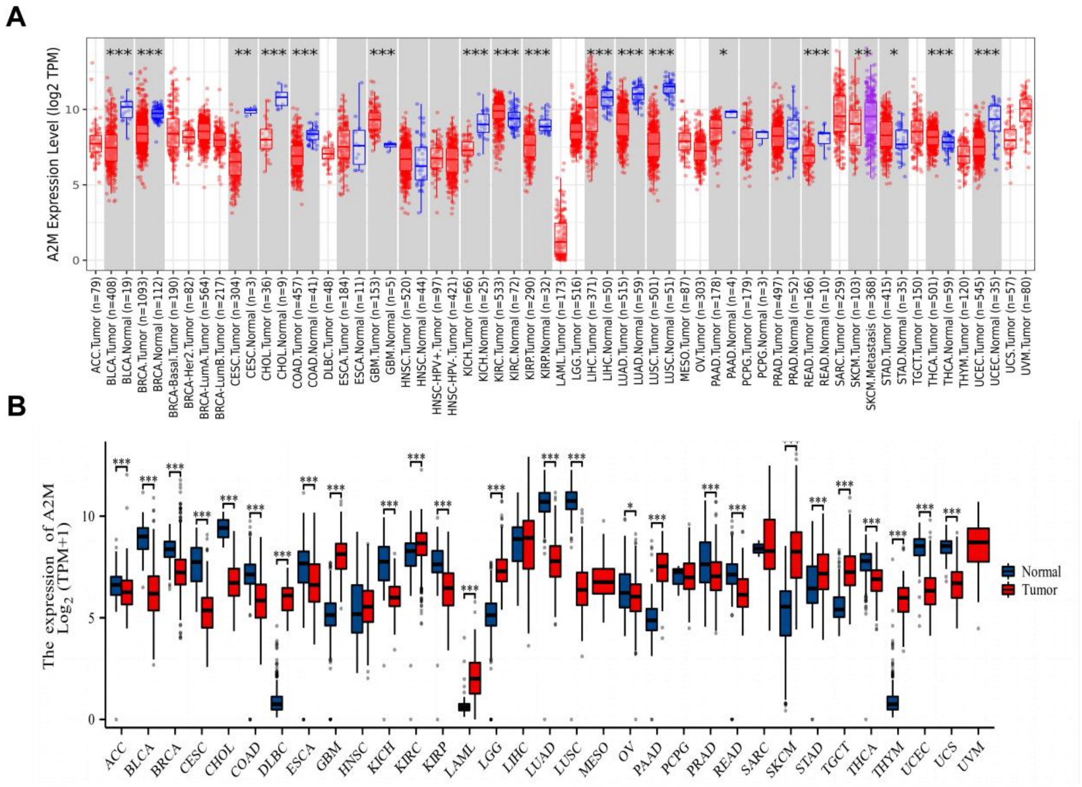

Figure S1

Differential expression of A2M in pancarcinoma; (A) Expression of A2M mRNA in pancarcinoma based on TIMER database; **(B)** Expression of A2M mRNA in pancarcinoma based on TCGA database; *: $p<0.05$ compare to normal; **: $p<0.01$ compare to normal; ***: $p<0.001$ compare to normal;

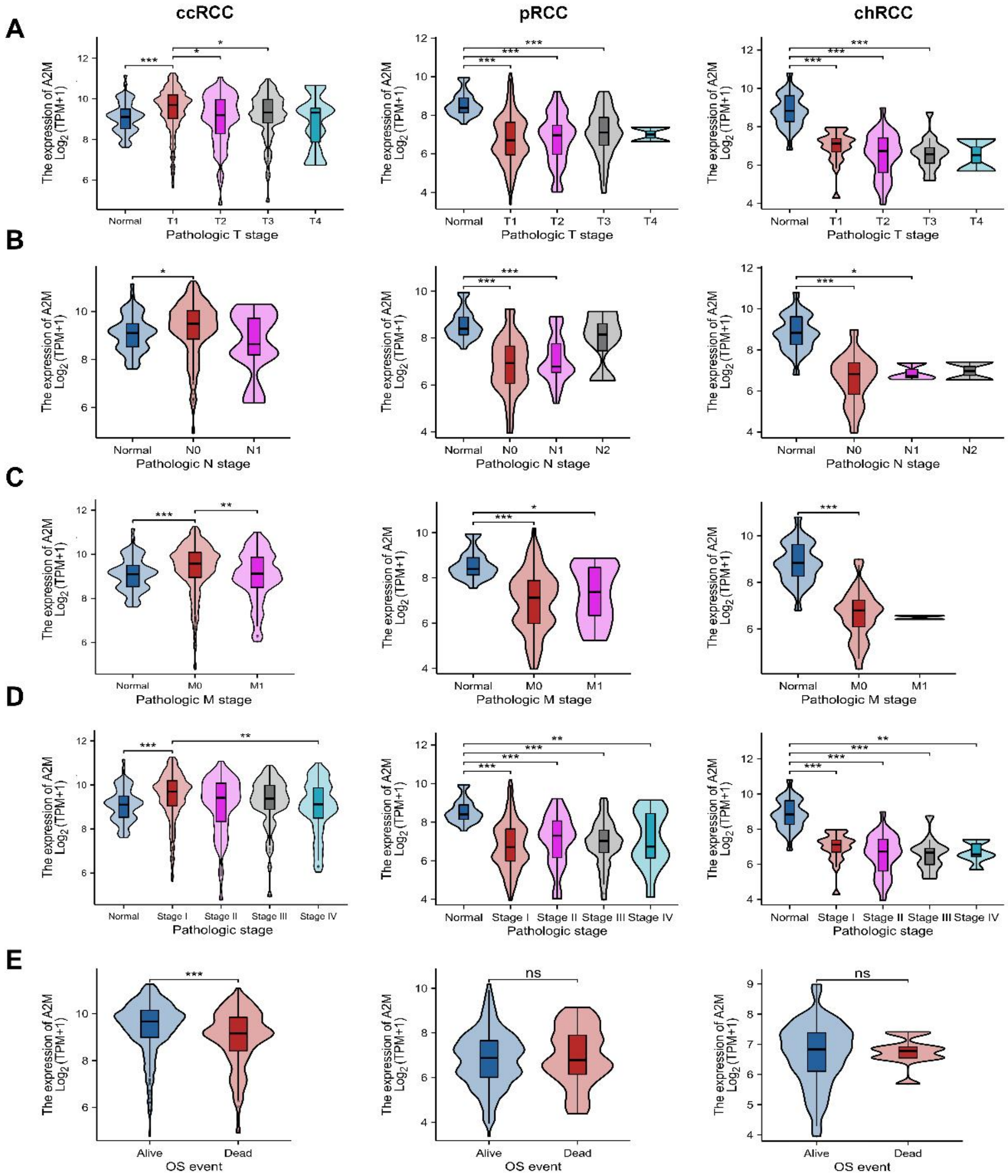

Figure S2

Association between A2M and clinical features and prognosis of RCC patients; (A-E) The differential expression of A2M in each clinical characteristic Stage (Pathologic T, Pathologic N, Pathologic M, Pathologic Stage, OS event) of ccRCC, pRCC and chRCC; *: p<0.05; **: p<0.01; ***: p<0.001;

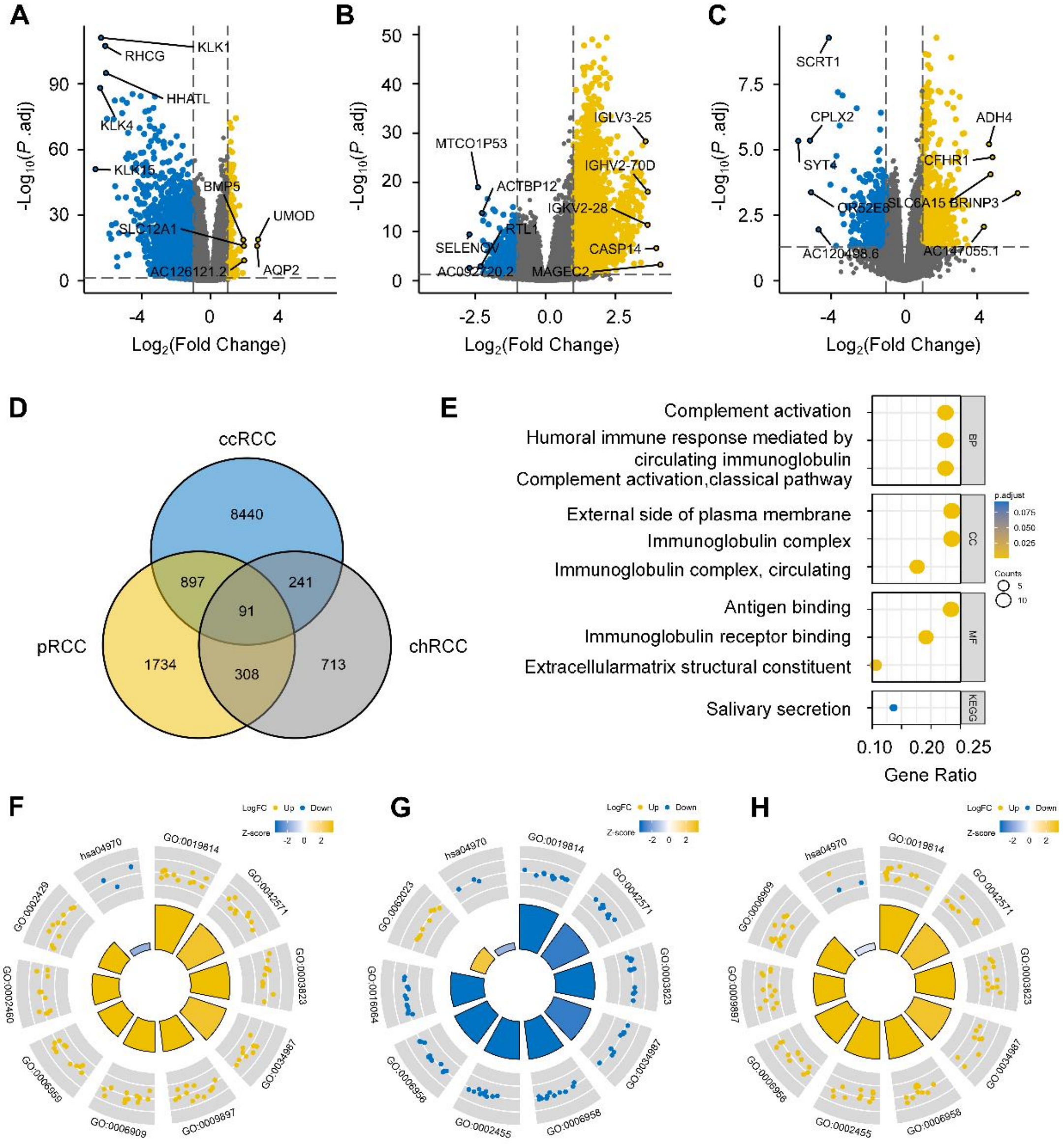

Figure S3

A2M is closely involved in immune regulation and response in the three common subtypes of kidney cancer; (A-C) Volcano maps based on the analysis results of A2M single gene differences in TCGA-KIRC, TCGA-KIRP and TCGA-KICH databases respectively (blue: down-modulated DEGs; Yellow: up-regulated DEGs; Gray: unchanged gene), and the first five genes with gene tags are the top five genes with the largest value of |log2FC| in the up and down regulated genes; **(D)** The intersection of DEGs (P < 0.05 and |log2FC| > 1) among the three tumor types was analyzed. There were 91 common genes in the three tumor types with the change of

A2M. **(E)** GO and KEGG enrichment analysis of co-DEGs; **(FGH)** Enrichment analysis of log2FC values of common differential genes (co-DEGs);

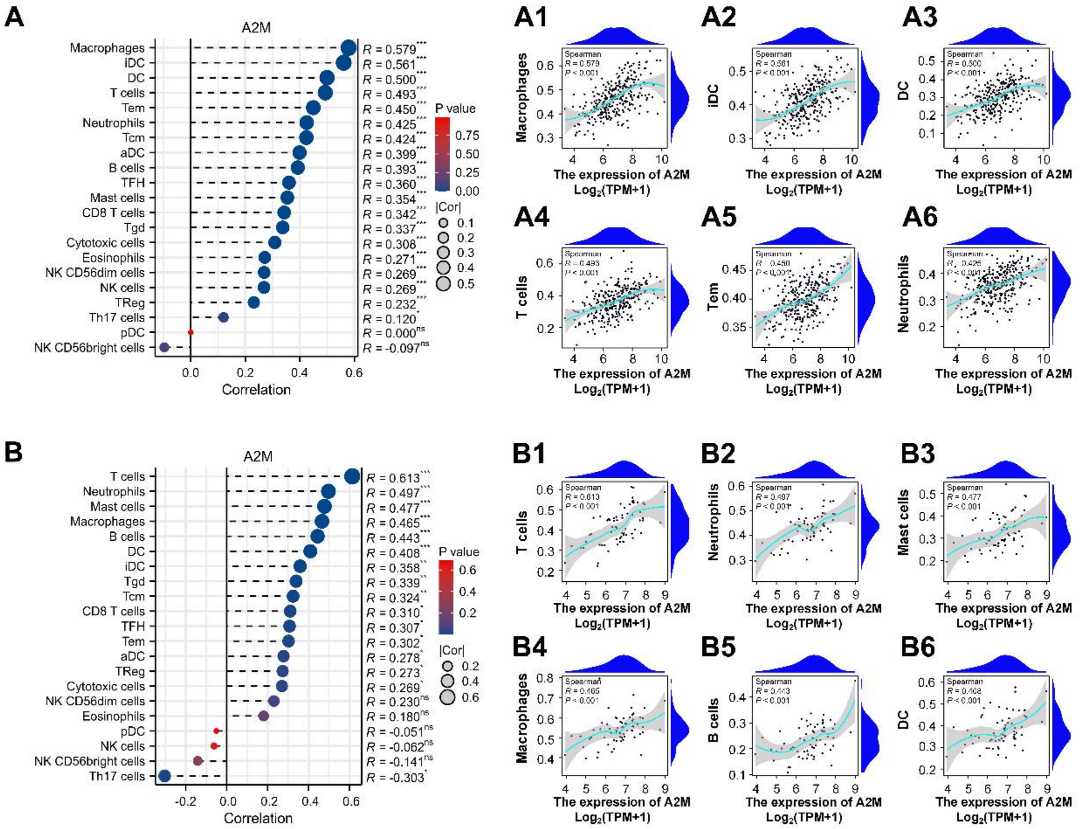

Figure S4

A2M is closely related to immune infiltration in pRCC and chRCC patients; (A) Correlation analysis of A2M expression in TCGA-KIRP and immune cell infiltration; **(A1-A6)** top6 immune cells in pRCC patients were closely related to A2M expression. **(B)** Correlation analysis of A2M expression in TCGA-KICH and immune cell infiltration; **(B1-B6)** top6 immune cells in chRCC patients are closely related to A2M expression;

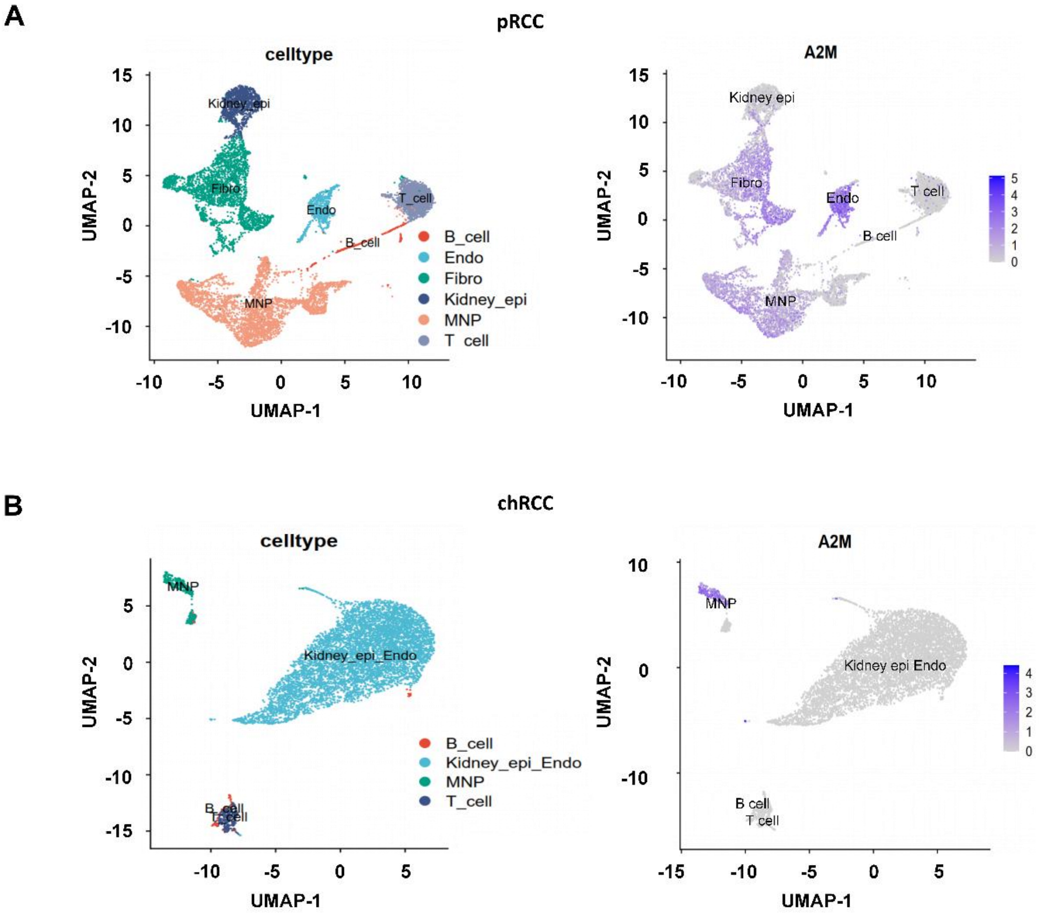

Figure S5

Single-cell analysis of A2M expression in pRCC and chRCC patients; (A) Single-cell analysis of the cell types of A2M expression in pRCC patients; **(B)** Single cell analysis of A2M expression in chRCC patients;

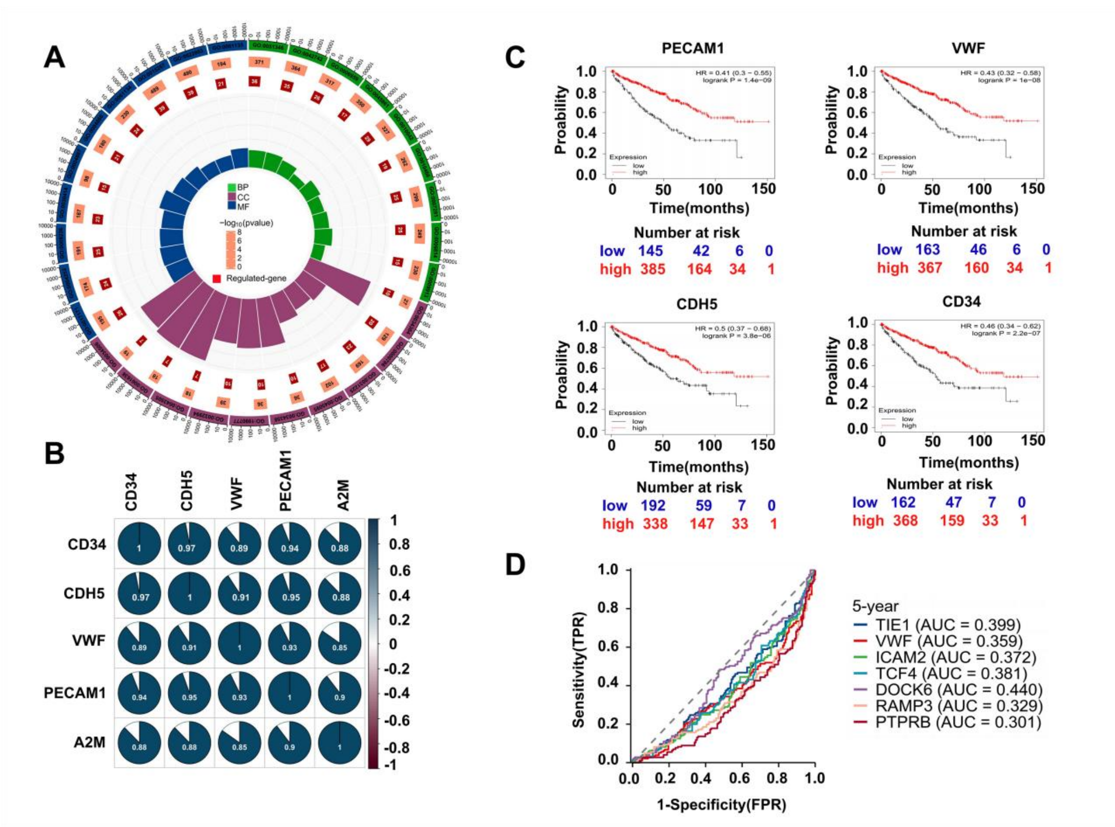

Figure S6

Go enrichment and survival-related analysis;

(A) GO enrichment analysis of DEGs in A2M single-gene analysis in TCGA-KIRC cohort (p < 0.05, |log2FC| > 1.5); **(B)** correlation coefficient diagram of A2M and 4 significantly interacting proteins in F; **(C)** Kaplan-Meier survival curve evaluation of the effects of CD34, PECAM1, CDH5, and VWF on the prognosis of ccRCC patients. **(D)** ROC analysis of disease prognosis of each model component gene;

Tables S1- S10

Table S1. Inclusion and exclusion criteria.

Overall Inclusion criteria

- Asian
- Age: 18–75years
- Gender：male and female
- Meeting the diagnostic criteria for clear cell renal cell carcinoma, papillary carcinoma and chromophobe renal carcinoma.
- Not receiving any form of antineoplastic therapy.
- Free of serious heart, liver, kidney and other important organ dysfunction, and no other kinds of or organ tumors.

Overall Exclusion criteria

- Received antineoplastic therapy.
- History of serious heart, liver, kidney and other important organ dysfunction, and no other kinds of or organ tumors.
- Psychosis or cognitive impairment, inability to properly understand trial objectives and risks.

Table S2. Clinical baseline data table of patients in the TCGA pRCC cohort.

Characteristic	levels	Low expression of A2M	High expression of A2M	p
n		145	146	
Clinical T stage, n (%)	T1	81 (77.9%)	58 (59.2%)	0.013
	T2	8 (7.7%)	19 (19.4%)	
	T3	15 (14.4%)	20 (20.4%)	
	T4	0 (0%)	1 (1%)	
	T Unknown	41 (NA)	48 (NA)	
Clinical N stage, n (%)	N0	72 (87.8%)	61 (84.7%)	0.855
	N1	9 (11%)	10 (13.9%)	
	N2	1 (1.2%)	1 (1.4%)	
	N Unknown	63 (NA)	74 (NA)	
Clinical M stage, n (%)	M0	41 (91.1%)	54 (91.5%)	1.000
	M1	4 (8.9%)	5 (8.5%)	
	N Unknown	100 (NA)	87 (NA)	
Primary therapy outcome, n (%)	PD	7 (6.3%)	0 (0%)	0.056
	SD	3 (2.7%)	2 (2.3%)	
	PR	2 (1.8%)	0 (0%)	
	CR	99 (89.2%)	86 (97.7%)	
	Unknown	34 (NA)	58 (NA)	
Clinical stage, n (%)	Stage I	80 (77.7%)	58 (60.4%)	0.005
	Stage II	4 (3.9%)	17 (17.7%)	
	Stage III	12 (11.7%)	17 (17.7%)	
	Stage IV	7 (6.8%)	4 (4.2%)	
	StageUnknown	42 (NA)	50 (NA)	
Age, n (%)	<=60	66 (45.8%)	69 (47.9%)	0.723
	>60	78 (54.2%)	75 (52.1%)	
	Unknown	1 (NA)	2 (NA)	

Table S3. Clinical baseline data table of patients in the TCGA chRCC cohort.

Characteristic	levels	Low expression of A2M	High expression of A2M	p
n		32	33	
T stage, n (%)	T1	6 (18.8%)	14 (42.4%)	0.218
	T2	14 (43.8%)	11 (33.3%)	
	T3	11 (34.4%)	7 (21.2%)	
	T4	1 (3.1%)	1 (3%)	
N stage, n (%)	N0	19 (86.4%)	20 (90.9%)	0.836
	N1	2 (9.1%)	1 (4.5%)	
	N2	1 (4.5%)	1 (4.5%)	
	N Unknown	10 (NA)	11 (NA)	
M stage, n (%)	M0	18 (90%)	16 (100%)	0.487
	M1	2 (10%)	0 (0%)	
	M Unknown	12 (NA)	17 (NA)	
Pathologic stage, n (%)	Stage I	6 (18.8%)	14 (42.4%)	0.212
	Stage II	14 (43.8%)	11 (33.3%)	
	Stage III	8 (25%)	6 (18.2%)	
	Stage IV	4 (12.5%)	2 (6.1%)	
Primary therapy outcome, n (%)	PD	2 (14.3%)	0 (0%)	0.008
	SD	1 (7.1%)	0 (0%)	
	PR	2 (14.3%)	0 (0%)	
	CR	9 (64.3%)	19 (100%)	
	Unknown	18 (NA)	14 (NA)	
Smoker, n (%)	No	20 (66.7%)	27 (93.1%)	0.012
	Yes	10 (33.3%)	2 (6.9%)	
	Unknown	2 (NA)	4 (NA)	
Age, n (%)	<=50	19 (59.4%)	14 (42.4%)	0.172
	>50	13 (40.6%)	19 (57.6%)	

Table S4. Clinical characteristics with the A2M expression based on 70 RCC patients in our institution

**Parameter**	**Type**	**A2m expression**								
		**ccRCC**			**pRCC**			**chRCC**		
		Low	High	p	Low	High	p	Low	High	p
Age	≤65	8(22.2%)	28(77.8%)	0.114	8(53.3%)	0	-	2(40.0%)	0	-
	>65	7(50.0%)	7(50.0%)		7(46.7%)	0		3(60.0%)	0	
Gender	female	4(25.0%)	12(75.0%)	0.843	4(26.7%)	0	-	2(40.0%)	0	-
	Male	11(32.4%)	23(67.6%)		11(73.3%)	0		3(60.0%)	0	
WHO/ISUP Grade	1-2	8(23.5%)	26(76.5%)	0.261	11(73.3%)	0	-	5(100%)	0	-
	3-4	7(43.8%)	9(56.3%)		4(26.7%)	0		0	0	
cT Stage	T1-2	12(80.0%)	32(20.0%)	0.506	12(80.0%)	0	-	5(100%)	0	-
	T3-4	3(91.4%)	3(8.6%)		3(20.0%)	0		0	0	
cN Stage	N0	9(22.5%)	31(77.5%)	0.054	13(86.7%)	0	-	5(100%)	0	-
	N1	6(60.0%)	4(40.0%)		2(13.3%)	0		0	0	
cM Stage	M0	10(23.8%)	32(76.2%)	0.077	13(13.3%)	0	-	5(100%)	0	-
	M1	5(62.5%)	3(37.5%)		2(86.7%)	0		0	0	

Table S5. GO enrichment analysis of A2M differential genes.

Ontology	ID	Description	GeneRatio	BgRatio	pvalue	p.adjust
BP	GO:0010817	regulation of hormone levels	35/766	496/18800	0.001195152	0.072836529
BP	GO:0051346	negative regulation of hydrolase activity	31/766	371/18800	0.000132536	0.018825079
BP	GO:0042742	defense response to bacterium	30/766	364/18800	0.000214687	0.025191294
BP	GO:0006959	humoral immune response	27/766	317/18800	0.000259323	0.028414361
BP	GO:0045861	negative regulation of proteolysis	27/766	350/18800	0.00118559	0.072836529
BP	GO:0016042	lipid catabolic process	26/766	327/18800	0.000939045	0.069254544
BP	GO:0010466	negative regulation of peptidase activity	25/766	262/18800	7.40953E-05	0.012867028
BP	GO:0007281	germ cell development	25/766	299/18800	0.000567672	0.049477808
BP	GO:0006814	sodium ion transport	24/766	249/18800	8.76127E-05	0.013999781
BP	GO:0009913	epidermal cell differentiation	21/766	230/18800	0.000491696	0.044896512
CC	GO:0034364	high-density lipoprotein particle	10/813	27/19594	6.35153E-08	2.6994E-05
CC	GO:0000786	nucleosome	20/813	129/19594	3.59258E-07	7.63423E-05
CC	GO:0031225	anchored component of membrane	23/813	169/19594	5.43163E-07	7.69481E-05
CC	GO:0045095	keratin filament	17/813	102/19594	9.49439E-07	9.65106E-05
CC	GO:0034358	plasma lipoprotein particle	10/813	36/19594	1.3625E-06	9.65106E-05
CC	GO:1990777	lipoprotein particle	10/813	36/19594	1.3625E-06	9.65106E-05
CC	GO:0032994	protein-lipid complex	10/813	39/19594	3.04357E-06	0.000184788
CC	GO:0043505	CENP-A containing nucleosome	7/813	18/19594	4.39604E-06	0.000207591
CC	GO:0061638	CENP-A containing chromatin	7/813	18/19594	4.39604E-06	0.000207591
CC	GO:0034506	chromosome, centromeric core domain	7/813	19/19594	6.71134E-06	0.000285232
MF	GO:0017171	serine hydrolase activity	26/706	195/18410	3.16345E-08	1.86094E-05
MF	GO:0004252	serine-type endopeptidase activity	24/706	174/18410	5.54677E-08	1.86094E-05
MF	GO:0008236	serine-type peptidase activity	25/706	191/18410	8.39685E-08	1.87809E-05
MF	GO:0030414	peptidase inhibitor activity	23/706	187/18410	8.42605E-07	0.000141347
MF	GO:0004867	serine-type endopeptidase inhibitor activity	15/706	98/18410	4.66704E-06	0.000626316
MF	GO:0004866	endopeptidase inhibitor activity	21/706	180/18410	5.84055E-06	0.000653168
MF	GO:0061134	peptidase regulator activity	24/706	230/18410	9.05663E-06	0.000868143
MF	GO:0015267	channel activity	39/706	489/18410	1.37732E-05	0.001075909
MF	GO:0022803	passive transmembrane transporter activity	39/706	490/18410	1.4431E-05	0.001075909
MF	GO:0061135	endopeptidase regulator activity	21/706	194/18410	1.8654E-05	0.001251681

Table S6. KEGG enrichment analysis of A2M differential genes.

ID	Description	GeneRatio	BgRatio	p.adjust
hsa05322	Systemic lupus erythematosus	20/277	136/8164	6.98328E-06
hsa05034	Alcoholism	21/277	187/8164	0.000168142
hsa04610	Complement and coagulation cascades	13/277	85/8164	0.000412796
hsa04613	Neutrophil extracellular trap formation	20/277	190/8164	0.000412796
hsa04966	Collecting duct acid secretion	7/277	27/8164	0.001255123
hsa04080	Neuroactive ligand-receptor interaction	28/277	362/8164	0.001486456
hsa00591	Linoleic acid metabolism	7/277	29/8164	0.001486456
hsa00980	Metabolism of xenobiotics by cytochrome P450	10/277	78/8164	0.009287266
hsa05204	Chemical carcinogenesis - DNA adducts	9/277	69/8164	0.014583016
hsa00982	Drug metabolism - cytochrome P450	9/277	72/8164	0.018038606
hsa00140	Steroid hormone biosynthesis	8/277	61/8164	0.023532816
hsa04024	cAMP signaling pathway	17/277	221/8164	0.02965382
hsa04950	Maturity onset diabetes of the young	5/277	26/8164	0.032239733
hsa04972	Pancreatic secretion	10/277	102/8164	0.044009713
hsa04976	Bile secretion	9/277	89/8164	0.054424979
hsa00590	Arachidonic acid metabolism	7/277	61/8164	0.0720495

Table S7. Top 15 up-regulated GSEA pathways of A2M differential genes.

Description	Enrichment Score	NES	pvalue	p.adjust
Lymph Angiogenesis Pathway	0.804380048	4.058054028	1E-10	2.32875E-08
S1P S1P3 Pathway	0.737139857	3.670626083	1E-10	2.32875E-08
Vegfr1 Pathway	0.699582727	3.609668294	2.49169E-10	4.22002E-08
Angiogenesis	0.787229012	3.53867214	1E-10	2.32875E-08
Mapk Targets Nuclear Events Mediated By Map Kinases	0.692109185	3.522074986	1E-10	2.32875E-08
Notch3 Intracellular Domain Regulates Transcription	0.691875692	3.490475611	1.57158E-09	1.8299E-07
Laminin Interactions	0.687290476	3.443400891	1E-10	2.32875E-08
Notch4 Intracellular Domain Regulates Transcription	0.79882981	3.401166645	1.84938E-10	3.82822E-08
Ncadherin Pathway	0.663523358	3.393786668	1E-10	2.32875E-08
Pdgfrbeta Pathway	0.66934304	3.333028322	1E-10	2.32875E-08
Pdgf Pathway	0.664711404	3.322100608	2.4561E-10	4.22002E-08
Vegf Pathway	0.665712562	3.27367462	1.29642E-09	1.72517E-07
S1P S1P1 Pathway	0.699586201	3.151719924	1.66384E-08	1.00505E-06
Nectin Pathway	0.626709383	3.139882954	8.8864E-10	1.37961E-07
Tgfb Pathway	0.732392778	3.116600425	1.29332E-08	8.60521E-07

Table S8. Top 15 down-regulated GSEA pathways of A2M differential genes.

Description	Enrichment Score	NES	pvalue	p.adjust
Apoptosis Induced Dna Fragmentation	-0.82845681	-1.904280038	1.25156E-05	0.000162864
Formation Of Senescence Associated Heterochromatin Foci Sahf	-0.787872828	-1.901785061	8.31295E-06	0.000118222
Dna Methylation	-0.657557146	-1.811188971	7.9175E-08	3.68757E-06
Assembly Of The Orc Complex At The Origin Of Replication	-0.648601395	-1.79503608	8.65156E-08	3.7946E-06
Sirt1 Negatively Regulates Rrna Expression	-0.649728421	-1.793893452	1.01197E-07	4.01127E-06
Regulation Of Gene Expression In Beta Cells	-0.693331067	-1.727749045	0.0007554	0.004675451
Reproduction	-0.600693162	-1.72730188	1E-10	2.32875E-08
Hcmv Late Events	-0.604325167	-1.723006779	1.42379E-09	1.76835E-07
Chylomicron Assembly	-0.794863246	-1.722361978	0.000942739	0.005505712
Linoleic Acid Metabolism	-0.663733712	-1.719625996	0.000219595	0.001690519
Amyloid Fiber Formation	-0.604711265	-1.718855367	2.22218E-09	2.30919E-07
Rmts Methylate Histone Arginines	-0.597311583	-1.671710945	2.11526E-06	4.06261E-05
Hnf3B Pathway	-0.613980039	-1.660661162	0.000106708	0.000951186
B Wich Complex Positively Regulates Rrna Expression	-0.590126694	-1.659455814	9.4976E-07	1.98809E-05
Base Excision Repair Ap Site_Formation	-0.601001002	-1.655409562	1.80118E-05	0.00021649

Table S9. Common immune characteristics of A2M in three renal carcinoma subtypes.

Ontology	ID	Description
CC	GO:0019814	immunoglobulin complex
CC	GO:0042571	immunoglobulin complex, circulating
CC	GO:0009897	external side of plasma membrane
BP	GO:0006958	complement activation, classical pathway
BP	GO:0002455	humoral immune response mediated by circulating immunoglobulin
BP	GO:0006956	complement activation
MF	GO:0003823	antigen binding
MF	GO:0034987	immunoglobulin receptor binding
BP	GO:0006909	phagocytosis
BP	GO:0006959	humoral immune response
BP	GO:0016064	immunoglobulin mediated immune response
BP	GO:0002460	adaptive immune response based on somatic recombination of immune recep tors built from immunoglobulin superfamily domains
BP	GO:0002429	immune response-activating cell surface receptor signaling pathway
CC	GO:0062023	collagen-containing extracellular matrix
KEGG	hsa04970	Salivary secretion

Table S10. GO and KEGG enrichment analysis of genes significantly related to A2M.

Ontology	ID	Description	GeneRatio	BgRatio	pvalue	p.adjust
BP	GO:0003158	endothelium development	8/39	133/18800	2.59558E-10	2.50473E-07
BP	GO:0045446	endothelial cell differentiation	7/39	117/18800	3.93467E-09	1.89848E-06
BP	GO:0045765	regulation of angiogenesis	8/39	345/18800	4.44283E-07	0.000121958
BP	GO:1901342	regulation of vasculature development	8/39	351/18800	5.06191E-07	0.000121958
BP	GO:0001570	vasculogenesis	5/39	80/18800	6.31908E-07	0.000121958
BP	GO:0035633	maintenance of blood-brain barrier	4/39	35/18800	7.90304E-07	0.000127107
CC	GO:0009897	external side of plasma membrane	7/40	455/19594	3.33968E-05	0.002658756
CC	GO:0005911	cell-cell junction	7/40	497/19594	5.84342E-05	0.002658756
CC	GO:0043296	apical junction complex	4/40	148/19594	0.000231188	0.007012691
CC	GO:0005923	bicellular tight junction	3/40	124/19594	0.002060164	0.044790406
CC	GO:0070160	tight junction	3/40	132/19594	0.002461011	0.044790406
CC	GO:0098636	protein complex involved in cell adhesion	2/40	43/19594	0.00348022	0.052783343
MF	GO:0098632	cell-cell adhesion mediator activity	3/40	54/18410	0.000218259	0.007222867
MF	GO:0005024	transforming growth factor beta-activated receptor activity	2/40	13/18410	0.000353639	0.007222867
MF	GO:0045159	myosin II binding	2/40	13/18410	0.000353639	0.007222867
MF	GO:0005178	integrin binding	4/40	156/18410	0.000357513	0.007222867
MF	GO:0098631	cell adhesion mediator activity	3/40	64/18410	0.000361143	0.007222867
MF	GO:0048185	activin binding	2/40	15/18410	0.000474745	0.007912425
KEGG	hsa04514	Cell adhesion molecules	7/19	157/8164	3.53221E-08	1.27159E-06
KEGG	hsa04670	Leukocyte transendothelial migration	4/19	114/8164	0.000118894	0.002140083
